# Exposure of Keratinocytes to *Candida Albicans* in the Context of Atopic *Milieu* Induces Changes in the Surface Glycosylation Pattern of Small Extracellular Vesicles to Enhance Their Propensity to Interact With Inhibitory Siglec Receptors

**DOI:** 10.3389/fimmu.2022.884530

**Published:** 2022-06-09

**Authors:** Adrian Kobiela, Joanna E. Frackowiak, Anna Biernacka, Lilit Hovhannisyan, Aleksandra E. Bogucka, Kinga Panek, Argho Aninda Paul, Joanna Lukomska, Xinwen Wang, Eleni Giannoulatou, Aleksandra Krolicka, Jacek Zielinski, Milena Deptula, Michal Pikula, Susanne Gabrielsson, Graham S. Ogg, Danuta Gutowska-Owsiak

**Affiliations:** ^1^ Experimental and Translational Immunology Group, Intercollegiate Faculty of Biotechnology of University of Gdansk and Medical University of Gdansk, University of Gdansk, Gdansk, Poland; ^2^ The Mass Spectrometry Laboratory, Intercollegiate Faculty of Biotechnology of University of Gdansk and Medical University of Gdansk, Gdansk, Poland; ^3^ State Key Laboratory of Military Stomatology, Department of Oral Medicine, School of Stomatology, The Fourth Military Medical University, Xi’an, China; ^4^ Medical Research Council (MRC) Human Immunology Unit, Medical Research Council (MRC) Weatherall Institute of Molecular Medicine, National Institute for Health Research (NIHR) Oxford Biomedical Research Centre, Radcliffe Department of Medicine, University of Oxford, Oxford, United Kingdom; ^5^ Computational Biology Research Group, Weatherall Institute of Molecular Medicine (WIMM), University of Oxford, Oxford, United Kingdom; ^6^ Laboratory of Biologically Active Compounds, Intercollegiate Faculty of Biotechnology of University of Gdansk and Medical University of Gdansk, University of Gdansk, Gdansk, Poland; ^7^ Department of Surgical Oncology, Medical University of Gdansk, Gdansk, Poland; ^8^ Laboratory of Tissue Engineering and Regenerative Medicine, Department of Embryology, Medical University of Gdansk, Gdansk, Poland; ^9^ Division of Immunology and Allergy, Department of Medicine Solna, Karolinska Institutet, Stockholm, Sweden; ^10^ Department of Clinical Immunology and Transfusion Medicine, Karolinska University Hospital, Stockholm, Sweden

**Keywords:** Candida albicans, glycosylation, extracellular vesicle, keratinocyte, siglec, atopic dermatitis (AD), immune evasion, exosomes

## Abstract

*Candida albicans (C. albicans)* infection is a potential complication in the individuals with atopic dermatitis (AD) and can affect clinical course of the disease. Here, using primary keratinocytes we determined that atopic *milieu* promotes changes in the interaction of small extracellular vesicles (sEVs) with dendritic cells and that this is further enhanced by the presence of *C. albicans*. sEV uptake is largely dependent on the expression of glycans on their surface; modelling of the protein interactions indicated that recognition of this pathogen through *C. albicans*-relevant pattern recognition receptors (PRRs) is linked to several glycosylation enzymes which may in turn affect the expression of sEV glycans. Here, significant changes in the surface glycosylation pattern, as determined by lectin array, could be observed in sEVs upon a combined exposure of keratinocytes to AD cytokines and *C. albicans*. This included enhanced expression of multiple types of glycans, for which several dendritic cell receptors could be proposed as binding partners. Blocking experiments showed predominant involvement of the inhibitory Siglec-7 and -9 receptors in the sEV-cell interaction and the engagement of sialic acid-containing carbohydrate moieties on the surface of sEVs. This pointed on ST6 β-Galactoside α-2,6-Sialyltransferase 1 (ST6GAL1) and Core 1 β,3-Galactosyltransferase 1 (C1GALT1) as potential enzymes involved in the process of remodelling of the sEV surface glycans upon *C. albicans* exposure. Our results suggest that, in combination with atopic dermatitis *milieu*, *C. albicans* promotes alterations in the glycosylation pattern of keratinocyte-derived sEVs to interact with inhibitory Siglecs on antigen presenting cells. Hence, a strategy aiming at this pathway to enhance antifungal responses and restrict pathogen spread could offer novel therapeutic options for skin candidiasis in AD.

## Introduction

*Candida albicans* (*C. albicans*) is a pathogen which can colonise the skin of atopic dermatitis (AD) patients, contributing to exacerbation of clinical symptoms (1, 2). Suspected mechanisms beyond the spread of the pathogen suggest that the exposure to *C. albicans* in the context of atopic inflammation promotes complex cytokine responses, with a pronounced involvement of Th17 cells (3–5), as confirmed by increased candidiasis risk in patients undergoing anti-IL-17 (6) therapy, in whom these cells are lacking. In effect, IgE-mediated hypersensitivity may follow (5), as a consequence of class-switching events (7) involving the antibodies directed against the yeast (1, 8). In addition, reduced lymphocyte proliferation upon *C. albicans* stimulation was observed in early AD studies (9); all this may compound the pathology.

*C. albicans* can be recognised by numerous innate receptors (10); epidermal keratinocytes, which form the uppermost layer of the skin and naturally come in contact with *C. albicans* are involved in the innate response directed against the fungus. Specifically, keratinocytes sense the invasion through pattern recognition receptors (PRRs) (11), i.e., Toll-like receptors (TLR) -2, -4 and -9, C-type lectins (dectin-1, DC-SIGN, mannose receptor), galectin-3 as well as NOD-like receptor NLRP3; some of those receptors are only expressed in activated keratinocytes and not in the steady state (11–14). Interestingly however, *C. albicans* has not been reported to be directly recognised by Siglec-type receptors which are abundantly expressed by the Langerhans cells. It has been documented that keratinocytes respond to the fungal threat by secretion of immune cell-attracting cytokines and chemokines (11).

In addition to the soluble factors, keratinocytes also secrete a very different kind of mediators, i.e. membranous organelles known as extracellular vesicles (EVs); of those, the fraction of exosome-enriched small EVs (sEVs) seems to be involved in long-distance communication. sEV membrane can either non-specifically fuse with the membrane of the recipient cell or participate in receptor-ligand interaction; both may result in the sEV uptake (15, 16). In addition, binding itself can impact processes occurring in the target cell (17, 18). Keratinocytes secrete sEVs (19–21) containing antigens that the cells are exposed to (19) and mediators which promote response against pathogens (22). Only one study so far investigated keratinocyte response to infection in the EV context; the authors showed that sEVs may be carriers for the staphylococcal enterotoxin and stimulate polyclonal T cell responses (22). Little is known on the role of keratinocyte-derived sEVs in the defense against other skin pathogens, including in atopic skin disease which predisposes to difficult to treat infections.

Here, we focused on the primary events at the initiation of the immune response, i.e. the process of sEV interaction with immune cells. We investigated the adhesiveness of sEVs secreted by keratinocytes during their differentiation process as well as the modifying effect of the AD *milieu* and exposure of the cells to common skin pathogens, i.e. *Candida albicans* and *Staphylococcus aureus*. Using lectin array we next profiled carbohydrate moieties present on the surface of the adhesive sEVs and identified glycosylation patterns which could be correlated with the increase in the propensity of sEVs to interact with dendritic cells (DCs). Modelling of those carbohydrate patterns onto DC receptors identified potential binding partners; these were validated experimentally. We found that Siglec-7 and Siglec-9 blockade reduced interaction of keratinocyte-derived sEVs, suggesting the role for those receptors in the process of information transfer between keratinocytes and antigen presenting cells, with relevance to the setting of allergic skin inflammation and *C. albicans* infection. Further analysis of the carbohydrate moieties suggested ST6 β-Galactoside α-2,6-Sialyltransferase 1 (ST6GAL1) and Core 1 β,3-Galactosyltransferase 1 (C1GALT1) as enzymes likely contributing to the changes on the sEV surface. Hence, targeting either this sialyltransferase or inhibitory Siglecs during sEV-cell interaction could be explored as a novel therapeutic strategy to enhance antifungal response in the patients.

## Results

### *Atopic* Dermatitis Inflammatory Milieu Promotes Changes *in sEVs* S*ecreted by* P*roliferatory and* Differentiated Keratinocytes Leading *to* Their Differential Interaction With Denritic Cells

**Figure 1:**
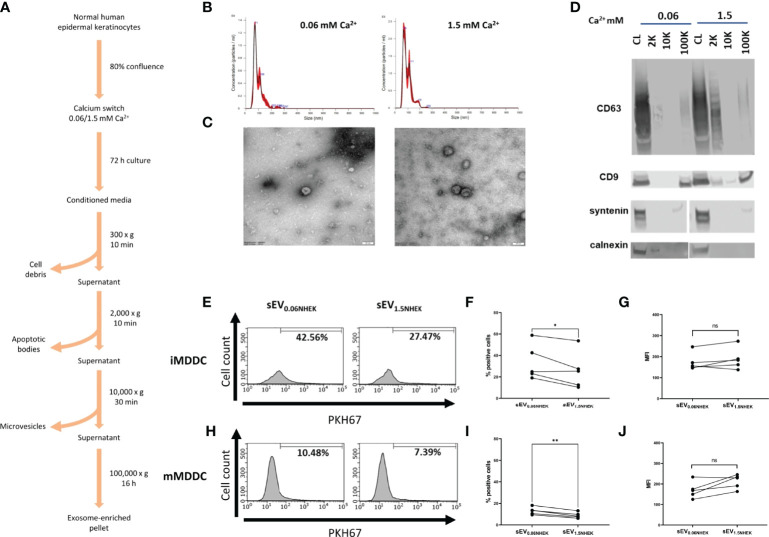
Atopic dermatitis *milieu* alters properties of keratinocyte-derived small extracellular vesicles (sEVs) which leads to their differential interaction with dendritic cells. **(A)** Protocol for sEV isolation from NHEK-conditioned medium; **(B)** size distribution of NHEK-derived sEVs (100K pellet) by Nanoparticle Tracking Analysis (NTA); **(C)** electron microscopy pictures confirming enrichment of cup-shaped exosomes within 100K pellet sEV fraction; scale bar: 50nm; **(D)** marker expression pattern in 2K, 10K and 100K fractions during isolation, confirming enrichment of exosomal makers in the sEV fraction (100K pellet); **(E)** iMDDC interaction with sEV_NHEK_ secreted under AD cytokine stimulation; example FACS data; **(F)** iMDDC interaction with sEV_NHEK_ under AD cytokine stimulation; % positive cells data pooled from n = 5 donors; **(G)** iMDDC interaction with sEV_NHEK_ under AD cytokine stimulation; MFI data pooled from n = 5 donors; **(H)** mMDDC interaction with sEV_NHEK_; example FACS data; **(I)** mMDDC % positive cells data pooled from n = 5 donors; **(J)** mMDDC interaction with sEV_NHEK_ under AD cytokine stimulation; MFI data pooled from n = 5 donors; *p < 0.05; **p < 0.01 (paired t-test). ns, not significant.

Keratinocyte gene expression is heavily remodelled during the process of differentiation and this affects all organelles (23). Keratinocyte-derived extracellular vesicles, and especially exosome-enriched sEVs may provide both an efficient source of antigenic information and contain innate mediators (24–26); their uptake may alert residing skin immune cells. Previous studies documented secretion of sEVs by epidermal murine and human keratinocytes (19, 20, 27, 28); however, little is known about functional effects of keratinocyte differentiation state on the fate of those vesicles and their downstream effect on immune responses to pathogens. Hence, we set out to investigate if the differentiation advancement of keratinocytes may have any effects on the process of sEV interaction with antigen presenting cells. To begin, we isolated primary normal human epidermal keratinocytes (NHEK) from skin samples. Cultures were brought to near-confluence; at this point we performed “calcium switch” on the cells, by either keeping them in the proliferation (low calcium; 0.06 mM) or differentiation-promoting (high calcium; 1.5 mM) medium. We harvested conditioned media after 72h, isolated sEV fractions (100K pellet) using ultracentrifugation method (**Figure 1A**) and assessed their size and morphology (**Figures 1B, C**). 100K pellets contained cup-shaped vesicles enriched in exosomal markers (CD63, CD9 and syntenin), while being negative for calnexin (**Figure 1D**), as expected (29).

Next, we set out to test for differentiation-dependent changes in the capacity of NHEK-derived sEVs to transfer the pathogen-dependent signals to antigen presenting cells. To this end, we generated dendritic cell models (iMDDC, immature dendritic cells and mMDDC, mature dendritic cells) and subjected them to the PKH67-labelled sEVs, to allow fluorescent tracking of their interaction with the cells by flow cytometry. To this end, since the technique itself does not allow to discriminate between the sEV binding and uptake, for the purpose of this study we interpret positive signal as the ‘interaction’ between sEVs and recipient cells. However, it is highly likely that the uptake may occur given the nature of these interactions. Here we found that both sEV_0.06NHEK_ and sEV_1.5NHEK_ interacted with mature and immature dendritic cells; the signal observed was higher for the latter, as expected given the efficient phagocytic ability of those cells. However, although we observed a trend towards more effective interaction of sEV_0.06NHEK_, this difference was not significant, suggesting that in the healthy skin this interaction is not dependent on the differentiation status of the secreting steady-state keratinocytes (**Figures S1A–F**). Nevertheless, we anticipated that keratinocytes might communicate *via* sEVs differently when activated through specific conditions, such as inflammation in AD. To test this we subjected the cells to the “AD cytokine cocktail” (containing IL-4, IL-13, IL-22 and TSLP) at the time of the calcium switch. Here, we observed that sEV_0.06NHEK_ were interacting more than sEV_1.5NHEK_ when produced by keratinocytes in the “AD inflammatory context”; the difference was not very pronounced, i.e. only 23% and 30% reduction for the imMDDC and mMDDC models, respectively; yet observed as significant and consistent for different donors (**Figures 1E–J**).

### *Keratinocyte-*D*erived sEVs* Are Enriched in Glycoproteins Involved in Adhesion

**Figure 2:**
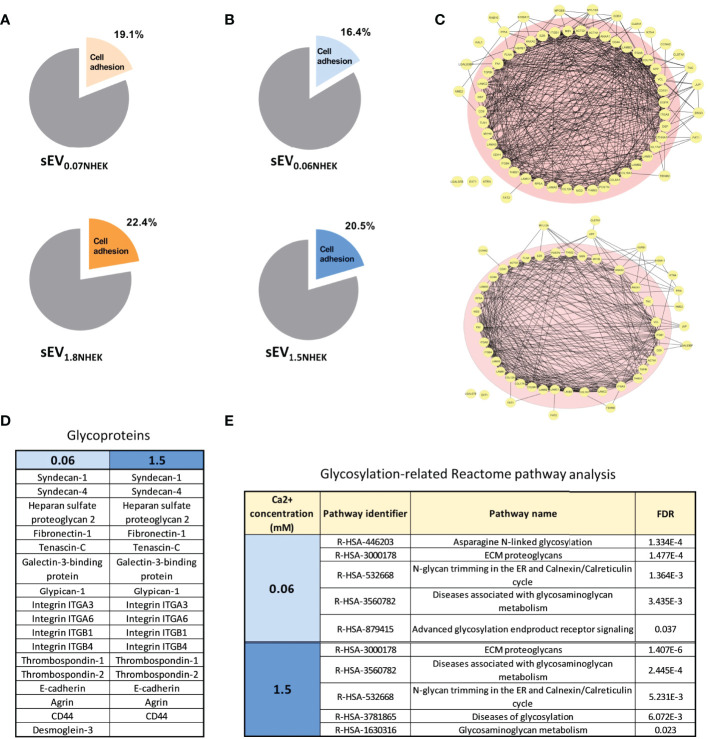
Keratinocyte-derived sEVs are enriched in glycoproteins involved in adhesion in steady state and in AD *milieu.* **(A)** Enrichment of adhesion proteins involved in cell adhesion in sEV_NHEK_ secreted in steady-state keratinocytes; Reactome - Gene Ontology terms re-analysis of the data available from Chavez-Muñoz et al. (28); **(B)** enrichment of adhesion proteins involved in cell adhesion in sEV_NHEK_ secreted by NHEKs exposed to AD *milieu* as identified by Reactome - Gene Ontology terms analysis; **(C)** interaction network for sEV adhesion-relevant proteins identified in sEV_0.06NHEK_ and sEV_1.5NHEK;_ **(D)** cell adhesion-relevant glycoproteins identified by mass spec in sEV_0.06NHEK_ and sEV_1.5NHEK;_ **(E)** Reactome-Gene Ontology identified term enrichment for the proteins in sEV_0.06NHEK_ and sEV_1.5NHEK_; FDR, False Discovery Rate; N.B. classical exosomal glycoprotein markers are included in the supplementary data (**Figure S2D**); mass spectrometry data based on n=3 biological replicates.

Next, to better understand how keratinocyte differentiation state in combination with AD *milieu* may affect the sEVs in the context of cell adhesion, we analyzed sEV_0.06NHEK_ and sEV_1.5NHEK_ proteomes by LC-MS/MS and further profiling using Gene Ontology (GO), STRING and Reactome Pathway Database. We first started with the re-analysis of the proteomic dataset published by Chavez-Muñoz et al. (28), which contained results of proteomic profiling of exosome-enriched sEVs (sucrose cushion purified) derived from NHEKs in a very similar model to ours (0.07 and 1.8 mM calcium was used in that study, similar to our work). These results suggested that during calcium-induced differentiation steady state cells increase content of sEV_NHEK_ proteins which could be assigned the ‘cell adhesion’ term (GO:0007155) by GO analysis, albeit this is not very pronounced (**Figure 2A**).

When we exposed sEV-secreting NHEKs to AD cytokines, we detected a greater variety of proteins in sEV_0.06NHEK_ in comparison to sEV_1.5NHEK_, suggesting that the differentiation process in keratinocytes leads to a shift to a more profiled sEV proteome (**Table S1**). Interestingly, all of the proteins identified in sEV_1.5NHEK_ were also found in sEV_0.06NHEK_. Similarly to the steady state conditions, we also observed a higher proportion of proteins assigned with the ‘cell adhesion’ term present in sEV_1.5NHEK_ compared to sEV_0.06NHEK_ (**Figure 2B**). Further STRING analysis of the ‘cell adhesion’-related proteins revealed strong predicted interactions between the vast majority of those (**Figure 2C**). A similar proportion of proteins among both sEV_0.06NHEK_ and sEV_1.5NHEK_ proteomes was predicted to interact with 10 or more partners within their corresponding datasets (pink circle in **Figure 2C**) and we did not observe any substantial differences between the conditions, suggesting that the presence of adhesion-relevant proteins alone in not sufficient to define the sEV_NHEK_ propensity for differential interaction with a cell. However, the adhesive properties of EVs have been shown to also depend on their surface glycosylation pattern. Hence, we next assessed the content of glycoproteins which may undergo such a modification. Nevertheless, the number of glycoproteins implicated in cell or extracellular matrix (ECM) adhesion detected in both sEV_0.06NHEK_ and sEV_1.5NHEK_ was similar (**Figure 2D**). Further analysis of the sEV_NHEK_ proteome against Reactome Pathway Database also revealed similar extent of overrepresentation in glycosylation-related pathways in both sEV_0.06NHEK_ and sEV_1.5NHEK_ (**Figure 2E** and **Figure S2A**). Interestingly, we also noted that a number of enzymes involved in protein glycosylation or N-linked carbohydrate processing during glycoprotein turnover were also present in sEV_0.06NHEK_ and sEV_1.5NHEK_ (**Figure S2B**).

### Exposure to *C. Albicans* but Not *S. Aureus* in the Context of AD Milieu Promotes sEV Cell Interaction

**Figure 3:**
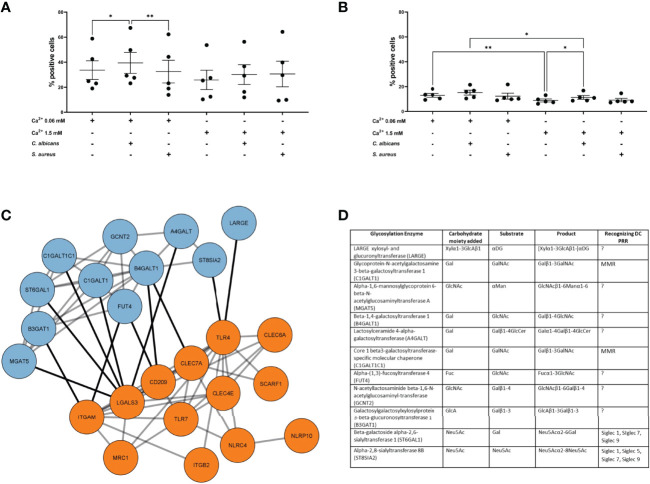
AD *milieu* and *C albicans* exposure affects sEV_NHEK_-cell interaction by interfering with glycosylation enzyme network in keratinocytes. **(A)** iMDDC interaction with sEV_NHEK_ under combined AD cytokine/pathogen stimulation; data pooled from n=5 donors; **(B)** mMDDCs interaction with sEV_NHEK_ under combined AD cytokine/pathogen stimulation; data pooled from n = 5 donors; *p < 0.05; **p < 0.01 (A-B: one-way ANOVA), means and SEM are shown; N.B. some comparisons with significant p-values are not labelled for the clarity of the graph; **(C)** STRING analysis of the protein networks between *C. albicans*-stimulated signalling from pattern recognition receptors in human keratinocytes (PRRs; in orange) and linked glycosylation-relevant enzymes (in blue); **(D)** Carbohydrate moiety/substrate/product-specificity of glycosylation enzymes identified in the network linked to *C. albicans*-specific PRRs (full list, including references in **Table S3**); DC, Dendritic cell; PRR, Pattern recognition receptor; PAMP, Pathogen associated molecular pattern; LacNAc, N-acetyllactosamine; GalNAc, N-acetylglucosamine; GlcNAc, N-acetylglucosamine; Fuc, Fucose; Gal, Galactose; Man, Mannose; Neu5Ac, N-acetylneuraminic acid; MMR, Macrophage mannose receptor; MGL, Macrophage galactose type lectin; DCIR, Dendritic cell immunoreceptor; BDCA2, Blood dendritic call antigen 2; CLEC, C-type lectin domain family; Siglec, sialic acid-binding immunoglobulin-type of lectin.

Since the previous results did not provide any strong indications on the functional differences we observed, i.e. sEV_NHEK_ secreted by both steady-state and “AD *milieu*-exposed” NHEKs seemed to have similar content of adhesion-relevant proteins, including glycoproteins, we deepened our analysis by the addition of another AD-relevant factor. Specifically, given that the allergic-type AD inflammation *milieu* is often clinically overlayed with an infection by AD-related pathogens, we aimed to investigate the effect of a combined stimulation of keratinocytes by AD cytokines and either *S. aureus* or *C. albicans* on sEV_NHEK_-cell interaction. Here, in the case of iMDDCs we noted increased interaction of the sEV_0.06NHEK_ secreted by AD/*C. albicans*-stimulated cells in comparison to the sEV_0.06NHEK_ obtained from keratinocytes treated only with the cytokines; no similar difference was noted for the sEV_1.5NHEK_ interaction (**Figure 3A**). In contrast, while differential interaction was also observed between AD/*C. albicans* vs AD-control in the mMDDC model, this was noted for sEV_1.5NHEK_ rather than sEV_0.06NHEK_ (**Figure 3B**). Intrestingly, we did not see any of those effects with sEVs produced upon the exposure of cells to AD/*S. aureus*, suggesting that the pathways which lead to the difference in the sEV interaction may be more specifically activated by the fungus and not broadly relevant to all skin pathogens or general keratinocyte activation.

### *Pathways of* Innate Recognition Of *C. Albicans and AD* Cytokine Signalling Are Linked *to the* Glycosylation Enzyme Network in Keratinocytes

Identification of additional conditions promoting sEV_NHEK_-cell interaction allowed us to further hypothesize on the mechanisms involved. Specifically, we asked whether *C. albicans* recognition by keratinocytes may affect pathways involved in glycosylation. To this end we next modelled protein networks between the pathogen recognition receptors (PRR) and glycosylation enzymes, based on the work of Schjoldager et al. (30) (**Table S2**). Given differential results between the two AD pathogens, for this analysis we only included PRRs known to be involved in the recognition of *C. albicans*, but excluded those implicated in the detection of *S. aureus*. The networks identified 11 enzymes with recognised links to PRR-mediated signalling (**Figures 3C, D**; full list, including references in **Table S3**), implying that exposure of keratinocytes to this pathogen may impact protein glycosylation.

### *Increased* Expression of Glycosylation Enzymes in the *AD* Skin Is Disease-Specific and Not Observed in Psoriasis

**Figure 4:**
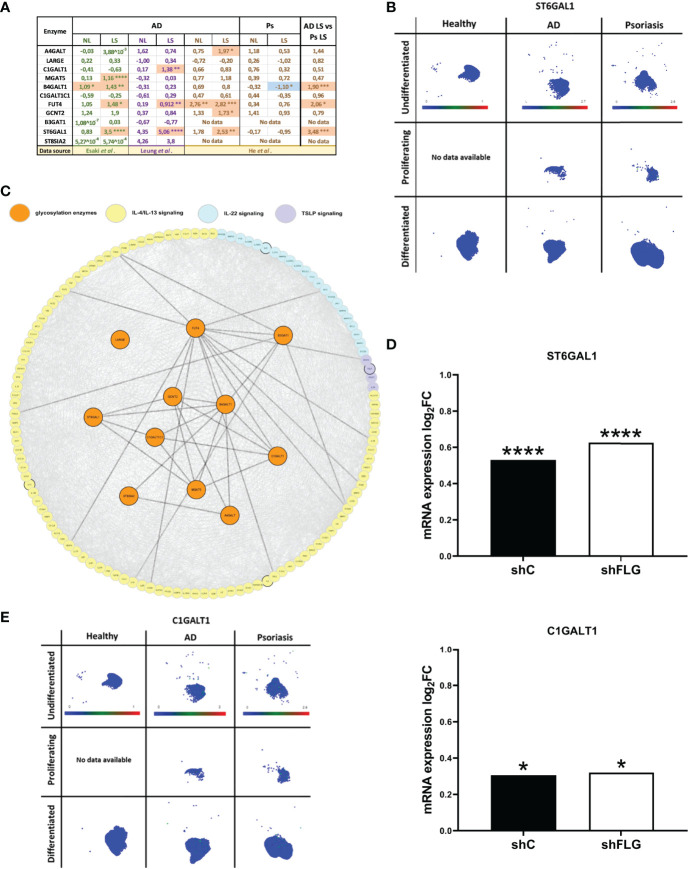
Glycosylation enzymes are upregulated in the epidermis of AD patients and keratinocytes exposed to AD *milieu.* **(A)** The expression of glycosylation enzymes in the epidermis of AD and Ps patients; analysis of the transcriptome profiling data available as datasets in Esaki et al., Leung et al. and He et al. (values in the table show expression log_2_FC compared to healthy epidermis); *p < 0.05; **p < 0.01; ***p < 0.001; ****p < 0.0001; (moderated t-test for Esaki et al. and He et al., multiple unpaired t-tests for Leung et al.); NL, non-lesional epidermis; LS, lesional epidermis; **(B)** UMAP plots of single cell expression of ST6GAL1 enzyme in keratinocyte subpopulations in the skin; data available through Human Developmental Cell Atlas; **(C)** protein network links between AD-relevant cytokine pathways and the previously identified 11 glycosylation-relevant enzymes; **(D)** mRNA expression of glycosylation enzymes differentially regulated upon exposure to IL-4/IL-13 stimulation in filaggrin-insufficient (shFLG; knockdown) keratinocytes and control (shC) keratinocytes (log2 fold change shown); combined data for n=3 biological replicates; *p < 0.05; ****p < 0.0001 (moderated t-test); **(E)** UMAP plots of single cell expression of C1GALT1 enzyme in keratinocyte subpopulations in the skin; data available through Human Developmental Cell Atlas; ST6GAL1, β-Galactoside α-2,6-Sialyltransferase 1; C1GALT1, Core 1 β,3-Galactosyltransferase 1.

Next, we investigated the levels of the identified enzymes in the AD skin, by analysing publically available transcriptome profiling datasets published by He et al., Leung et al. and Esaki et al. (31–33). This analysis revealed differential expression for 7 out of 11 glycosylation enzymes listed in **Figure 3D**. Specifically, while the identified proteins differed between the studies, we consistently noticed a positive change in expression (upregulation) for all the differentially regulated genes in AD; this was observed both in the epidermal samples obtained by tape stripping (He et al. and Leung et al.) and those harvested by laser-capture microdissection (Esaki et al.), increasing our confidence in the physiological relevance of the obtained data. Here we noted several enzymes differentially expressed, however, only FUT4 and ST6GAL1 were detected as upregulated in at least two of those datasets (**Figure 4A**). Furthermore, a comparison of the levels of the enzymes in AD vs psoriatic epidermis suggested a degree of disease-specificity, with B4GALT1, FUT4 and ST6GAL1 found expressed at levels significantly higher than in psoriasis. Interestingly, of all the enzymes, the highly upregulated expression of ST6GAL1 in lesional AD epidermis compared to the healthy epidermis was the most consistent and significant across all the three datasets analyzed. However, the single cell dataset that we sourced from the Skin Cell Atlas (34) was somehow different, showing increased expression in both those inflammatory skin diseases (**Figures S5A, B**), including in proliferating, undifferentiated and differentiated keratinocytes (**Figures 4B**, **S5B**).

### *Upregulation of* Glycosylation Enzymes in the *AD* Skin Is Driven by Atopic Milieu and Not by Filaggrin Insufficiency

Next, having gathered substantial AD-relevant evidence, we aimed to provide additional insights into the causes leading to the expression changes we next attempted to identify links between the enzymes and signalling networks of AD cytokines (**Figure 4C**). This highlighted several connections to their downstream pathways which may suggest potential modulatory effect on the glycosylation network. To this end, we noticed that those connections were present mainly downstream from the IL-4/IL-13 pathway. To this end, we identified a transcriptomic dataset which reported an increase in expression of ST6GAL1 in normal keratinocytes (reconstructed into an organotypic model) upon IL-4/IL-13 treatment (35), further suggesting that atopic cytokines may affect this pathway.

Downregulation of filaggrin (FLG) in AD skin is one of the hallmarks of the disease; the extensive impact of this downregulation is a reflection of multifaceted role FLG carries out in the skin (36, 37), also at the keratinocyte biology level. Hence, it is plausible that FLG insufficiency itself could also affect the expression of the glycosylation enzymes; in which case the effect in the skin would not be exclusive to the *milieu* but could potentially require the underlying FLG insufficiency background providing synergestic effect. To test this, we used filaggrin-insufficient keratinocytes (shFLG), in which FLG expression was reduced by shRNA interference (38–40) and the control (shC) line; we assessed mRNA expression in both lines upon exposure to IL-4/IL-13. We determined that two of the enzymes, i.e. ST6 β-Galactoside α-2,6-Sialyltransferase 1 (ST6GAL1) and Core 1 β,3-Galactosyltransferase 1 (C1GALT1) were upregulated by this treatment (**Figure 4D**); expression of both of those enzymes was increased in AD epidermis as detected before in AD skin. Importantly, however, there was no difference between shC and shFLG cells, ruling out that FLG status is prerequisite to the observed effect. Given that C1GALT1 is a core extension enzyme and its activity in generating O-linked glycans is prerequisite to the action of the ST6GAL1 (as a capping enzyme), we also investigated the distribution of its expression reported by Skin Atlas. Here, we noted that C1GALT1 levels are pronouncedly upregulated in both AD and psoriatic keratinocytes, with no visibly significant difference between the two conditions (**Figure 4E**), which is in line with the analysis of the skin transcriptomic data (**Figure 4A**). This may suggest that the expression of the two enzymes may be regulated differently with the involvement of either both C1GALT1 and ST6GAL1 or exclusively ST6GAL1 for AD and psoriasis, respectively.

### *sEVs* Secreted Under Exposure to *C. Albicans* and *AD* Cytokines Express Altered Surface Glycosylation Pattern

**Figure 5:**
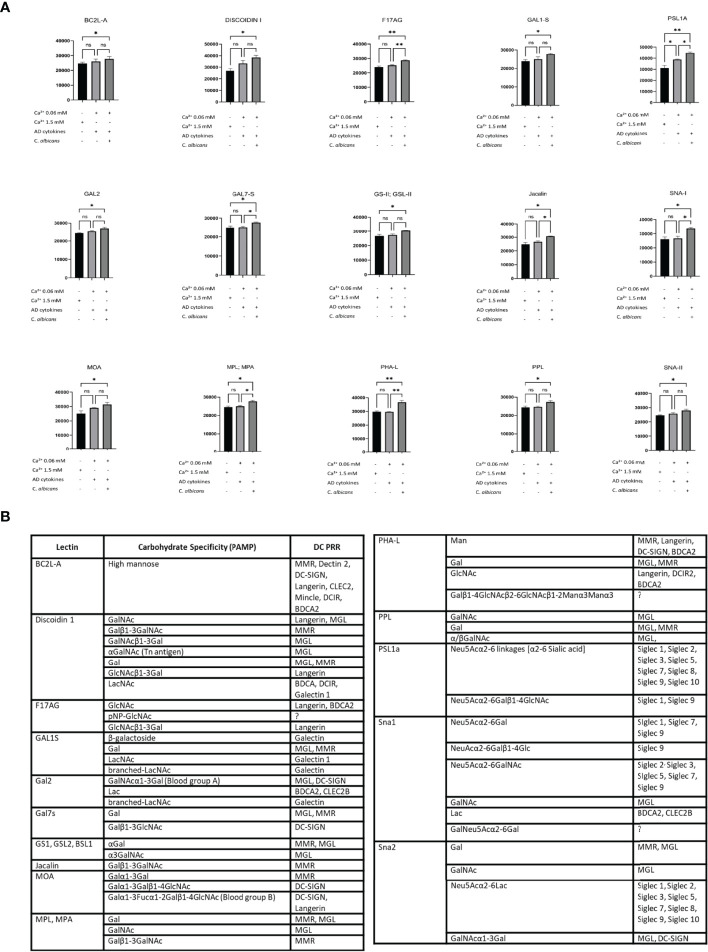
Lectin array-based identification of carbohydrate moieties on the adhesive sEV_NHEK_ map to potential receptors on antigen presenting cells. **(A)** Lectin binding to carbohydrate moieties on the surface of sEV_NHEK_; data summarizes biological duplicates with n=3 keratinocyte donors; *p < 0.05; **p < 0.01; **(B)** carbohydrate moieties enriched in the more adhesive sEVs as identified by lectin array, together with potential binding receptors on antigen presenting cells as identified by the literature search (full list, including references in **Table S4**). ns, not significant.

Next, to investigate the glycosylation pattern on the surface of sEVs which were characterised by increased cell interaction, we proceeded with the identification of the sEV membrane-exposed carbohydrate moieties by lectin array. Lectin array is a useful tool for glycosylation pattern identification; lectins on the slide have binding specificity towards defined carbohydrate moieties which allows identification of glycosylation of the bound molecules (either soluble or displayed on sEV (41)). To dissect the differences in the functional outcomes, we selected conditions on a spectrum of the interaction characteristics, i.e. AD/*C. albicans* sEV_0.06NHEK_ vs AD cytokines sEV_0.06NHEK_; we also included the condition which resulted in the lowest level of this interaction observed in our experiments, i.e. sEV_1.5NHEK_ (at steady state). We observed substantial binding of sEVs to the array for 17 out of 70 lectins reporting significant changes between the conditions (**Figure 5A**, **Figure S2C**); we identified enrichment rather than *de novo* appearance of any additional carbohydrate patterns on sEVs secreted upon the combined AD/*C. albicans* stimulation. The results were filtered to identify lectins for which sEV binding to the array followed the functional results of the cell interaction (the highest for the AD/*C. albicans* sEV_0.06NHEK,_ the lowest for sEV_1.5NHEK_ and intermediate for AD cytokines sEV_0.06NHEK_). This yielded 15 lectins binding glycans enriched in sEVs characterised by increased interaction; the binding from the majority (almost 90%) of the lectins with differential outcomes represented the trend, indicating unidirectional alterations in the sEV glycan profile. The resulting panel of the identified carbohydrate moieties was next matched to the innate carbohydrate recognition receptors on the antigen presenting cells, yielding several potential binding partners suitable for experimental validation (**Figure 5B**; full list, including references in **Table S4**).

### *Siglec-7 and Siglec-9* Receptors Are Involved in the Interaction Between Keratinocyte-Derived sEVs and Antigen Presenting Cells

**Figure 6:**
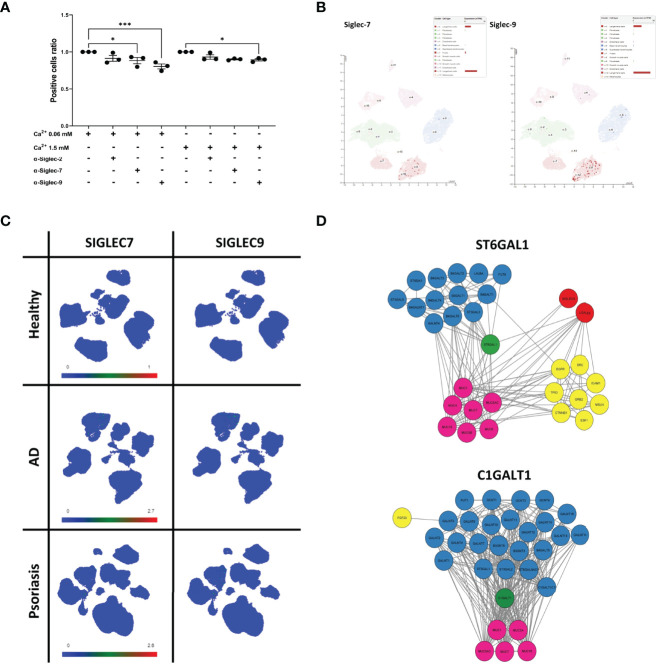
Sialyltransferase ST6GAL1 is implicated in sialylation pattern on keratinocyte-derived sEVs and defines their cell interaction propensity *via* Siglec-7 and Siglec-9 receptors. **(A)** Identification of binding receptors for sEV_N/TERT-1_ cell interaction by blocking experiments with anti Siglec-type receptors on differentiated THP-1 cells; combined data for n=3 biological replicates; **(B)** single-cell RNA expression levels of Siglec-7 and Siglec-9 in the epidermis of the skin; data available through ProteinAtlas; **(C)** UMAP plots of single cell expression of SIGLEC7 and SIGLEC9 receptors in the skin; data avalabale through Human Developmental Cell Atlas; **(D)** protein interaction network of ST6GAL1 and C1GALT1 enzymes obtained *via* STRING analysis. * - p<0.05, *** - p<0.001.

Since the binding to the array revealed a considerable level of detected glycans on sEV_NHEK_, we reasoned that these glycosylation patterns may also promote interaction between sEVs and antigen presenting cells in the steady state. Hence, to identify the receptor route involved, we next performed receptor blocking experiments, using IL-4/GM-CSF-differentiated THP-1 cells, serving as antigen presenting cells and N/TERT-1 immortalised keratinocytes (42, 43) as an efficent sEV source (**Figure S3**). Given the previously identified carbohydrate moieties involved, we focused on the C-type Lectin Receptors (CLRs), i.e. Langerin (CD207), Macrophage Mannose Receptor (MMR; CD206), Dendritic Cell-Specific Intercellular adhesion molecule (DC-SIGN; CD209). In addition, we also investigated Sialic acid-binding Immunoglobulin-like Lectins known to recognize sialic acid. Specifically, we included Siglec-2, Siglec-7 and Siglec-9, as these receptors could be matched to the recognized carbohydrate moieties detected by the array but they differ in specificity and affinity to the same glycans (44). In this model differences could be observed in the expression level for the selected receptors during the differentiation process; a positive population was present for each of the markers and further increased during THP-1 differentiation (**Figure S4A**). Of those, DC-SIGN expression was the highest and seen for nearly all of the cells; on the other hand, Langerin was the least abundant marker, as expected, but also showed an increase in expression. However, the differences in the outcome of the blocking experiments with specific antibodies could not be simply explained by the variation in the receptor expression levels. Specifically, while we observed no clear effect of the anti-MMR, -Langerin, -DC-SIGN (**Figure S4B**) and Siglec-2 antibodies, blocking with Siglec-7- and Siglec-9-specific antibodies significantly decreased cell interaction of either sEV_0.06NHEK_ or both sEV_0.06NHEK_ and sEV_1.5NHEK_, respectively (**Figure 6A**); the most profound effect was observed upon Siglec-9 blockade. We also observed the p-value approaching significance (p=0.063) for the anti-Siglec-7 blocking of the sEV_1.5NHEK_ interaction; as well as (a non-significant) trend for anti-Siglec-2 blokade. Siglec-7 and Siglec-9 are known inhibitory receptors which decrease PRR-dependent activation of the cell upon sialic acid binding (45, 46). Their expression within the epidermis is almost exclusively confined to the population of Langerhans cells (**Figure 6B**). In the whole skin samples those receptors have broader and elevated expression in inflammatory skin diseases, i.e. AD and psoriasis (Ps). However, the expression is, still mainly confined to the myeloid cell populations, as identified by single cell analysis available through Skin Cell Atlas (34). This includes macrophages, monocyte-derived DCs, LCs, etc., which can serve as antigen presenting cells in the skin (**Figure 6C**; **Figure S5A**). Combined, these results suggest that sialic acid moieties could provide a specific targeting motif directing keratinocyte-derived sEVs to antigen presenting cell populations in the tissue.

### *Changes in* Glycosylation Pathways Are Implicated *in* Sialylation Pattern *on* Keratinocyte-Derived *sEVs and* Define Their Interaction With *Siglec-7 and Siglec-9* R*eceptors*

Finally, we set out to determine the specific enzyme(s) implicated in the observed process of sEV surface glycan remodelling. Indeed, the ST6GAL1 enzyme identified by us earlier within the glycosylation enzyme network linked to the *C. albicans*-stimulated PRR signalling (**Figure 3C**) and most consistently upregulated in AD skin and keratinocyte cultures by IL-4/IL-13 (**Figure 4**) was an immediate match to the glycosylation pattern observed, as it catalyses the reaction of the addition of sialic acid *via* an α2-6 linkage (**Figure S6A**). The associated GO terms for this enzyme reflected as enrichment of the term GO: 0005515 “Enables protein binding” (**Figure S6B**). We were not able to match other enzymes which we found upregulated by AD cytokines or in patient epidermal samples, including FUT4 enzyme, highly differentially expressed and consistent between the three studies. Likewise, C1GALT1 also does not seem to be of the primary importance in our model given that it despite that it produces Galβ1-3GalNAc linkage detected in the array; however, this linkage is recognised by the MMR receptor (47), blockade of which did not affect the strength of sEV-cell interactions (**Figure S4B**).

Finally, with the aim of identifying sEV_NHEK_ proteins that could be targeted by glycosylation changes we carried out further analysis, focusing on the direct interactomes of ST6GAL1 and C1GALT1 (**Figure 6D**). This revealed links to the other glycosylation-relevant proteins. In addition, both enzymes link to several mucins as expected given their inclusion in the mucin formation pathways. In addition, a node connection between ST6GAL1 and galectin-3 (LGALS3) can be also seen. This suggests potential substrates for the enzymes, displayed on the sEV_NHEK_ surface; unfortunately, none of those were found in our mass spectometry dataset, thus not providing us with any indications regarding the identity of the potential targets.

## Discussion

Small extracellular vesicles (sEVs) produced by virtually all living cells, including those of non-immune origin have been shown to participate in immune responses, both innate and adaptive (48, 49). sEV-specific role depends on their capacity to directly interact with cells, where receptor-ligand interactions could lead to a downstream effect. This cellular interaction is also critical initial event enabling sEV uptake and intracellular transfer of sEV cargo, enriched in antigens and innate signalling molecules or other immune-relevant compounds. Experiments with various cell lines indicated that sEV glycan composition, which is important during cellular adhesion of sEVs, is dependent on the cellular source. Importance of glycans as potential targeting motifs for the recipient cell was previously indicated (50).

To this end, our results suggest that the exposure of keratinocytes to stimulation relevant to atopic dermatitis (AD), i.e. allergic inflammatory *milieu* and *C. albicans* may induce relevant changes in the sEV surface glycosylation patterns and are translated into differential functional outcomes; here we showed certain increase in the propensity for sEV interaction with dendritic cells. By carbohydrate moiety identification with lectin array we subsequently determined that these alterations include enhanced expression of forms of sialic acid. Modelling indicated that the effect downstream of the PRR signalling induced by *C. albicans* on the activity of sialyltransferase ST6GAL1 could provide a plausible explanation for this effect. Sialic acid-containing motifs were abundant on keratinocyte-derived sEVs and enriched further in vesicles characterised by enhanced propensity for cell interaction (41).

Innate recognition of sialic acid is mediated by a group of Sialic acid-binding Immunoglobulin-like Lectins (Siglecs) and we determined that the cell interaction of keratinocyte-derived sEVs in our model is Siglec-7 and Siglec-9-mediated. Furthermore, the dependence on either both Siglecs or exclusively Siglec-9 for the undifferentiated vs. differentiated keratinocytes as sEV sources, respectively, mirrors the difference observed by us earlier at the functional level. Specifically, higher interaction efficiency could be observed for sEV_0.06NHEK_, for which the interaction seems to be aided by both of these receptors. Being two members of the CD33-related Siglec family both Siglec-7 and Siglec-9 are considered “endocytic” receptors (51, 52); therefore the sEV binding may promote their efficient uptake by the cells expressing those receptors. From the first glance this could appear as benefiting the host, since provision of *C. albicans* antigens and other stimulatory signals within the cargo should promote T cell responses. However, it has been shown that signalling through Siglec-7 and Siglec-9, which contain tyrosine-based inhibition motif (ITIM) within their intracellular domains dampens proinflammatory responses by inhibiting NF-κB-dependent TLR4 signalling pathway (53); as a result, stimulation of these “inhibitory Siglecs” provides strong negative signal. Interestingly, data published by Varchetta et al. (54) stands in contrast to the studies implicating the inhibitory role of Siglec-7 in immune response, suggesting a potential role of sialic acid-*independent* stimulation of this receptor in triggering the release of proinflammatory cytokines by monocytes. In this particular study Siglec-7 was activated by either antibody-mediated crosslinking or zymosan, a yeast cell wall-derived particle devoid of any forms of sialic acid. Here, since we propose a mechanism critically dependent on the sialic acid engagement of Siglec-7, the study might be less relevant in the context of our findings. However, it brings up a potentially important question regarding the possibility of different functional outcomes upon the activation of the Siglec-7 pathway depending on the chemical composition of the ligand. Furthermore, while we did not establish the content of the sEV_NHEK_ cargo, these may contain RNA species, incl. regulatory, proteins and other effector molecules which could potentially also contribute to the effect.

While *C. albicans* expresses several PRR agonists, it does not seem to express enough sialic acid to stimulate Siglec receptors (10) and switch off the NF-κB signalling by Siglec-dependent inhibition. Besides, as a predominantly intracellular pathogen, *C. albicans* may not have any effective means to directly interact with the membrane-expressed Siglecs on the antigen presenting cells. Hence, the proposed mechanism could potentially increase the chances of the pathogen to achieve the induction of tolerogenicity, if *C. albicans* exploit the host’s enzymatic machinery to induce sialic acid coating on sEVs. It has been also demonstrated that antigen sialylation results in the inhibition of Th1 and Th17 cells and induction of Treg subsets (55); given the Th1/Th17-dominated effective antifungal response, such an effect would similarly benefit *C. albicans*. Intrestingly, the inhibitory Siglec pathways seem to be hijacked by numerous PRRs-stimulating pathogens escaping immunosurveillance (46). To our best knowledge we are the first to report a possibility of remodelling of the host sEV surface glycosylation by a pathogen which could also constitute a potentially attractive and resource-saving immune evasion strategy. However, the relevance of this mechanism would have to be confimed in the dedicated immune evasion *in vivo* studies.

In the context of the skin disease, this mechanism may potentially have an important effect on the Siglec-7 and Siglec-9 expressing Langerhans cells (LCs). LCs switch between immunomodulation and immunactivation by integration of incoming stimuli (56); in the healthy skin this would mean tolerogenic phenotype upon encounter of non-threatening signals and proinflammatory one during skin infection. By preventing LC activation with inhibitory signalling, *C. albicans*-induced sEV sialylation could target those cells predominantly, given the LC-exclusive expression of these Siglecs in the epidermis. Deeper in the tissue, these receptors are also expressed by additional myeloid cells, all of which could both present antigens and react to innate stimuli, e.g. macrophages or dendritic cells. Hence, sialic acid-dependent exosome/sEV-mediated activation inhibition could have a potential to affect clearance of *C. albicans* from the AD skin and lead to its enhanced spreading.

Recently, the inhibitory Siglec-type receptors were proposed as a novel class of immune checkpoints targeting myeloid cells with inhibitors suggested for clinical application (45). In addition, in the cancer setting T cells may also express inhibitory Siglec-7 and Siglec-9, meaning that they could be directly targeted; in agreement with this, sialic acid-dependent exosome/sEV-mediated direct T cell inhibition was also previously shown (57). Interestingly, candidiasis itself increases risk of many malignancies (58); hence, the question that needs further addressing is if *C. albicans*-remodelled sEVs could suppress anticancer response and promote tumour growth.

Mammalian proteins involved in the glycosylation processes show great diversity (30). In our study, we identified 11 enzymes which could be linked to the innate response to *C. albicans* in keratinocytes. Of those, ST6 β-Galactoside α-2,6-Sialyltransferase 1 (ST6GAL1) and Core 1 β,3-Galactosyltransferase 1 (C1GALT1) seemed to be involved in the changes in the glycosylation pattern induced by the fungus on keratinocyte-derived sEVs, i.e. both enzymes are upregulated in AD and Ps and expressed in the majority of skin cell populations, including all subopulations of (proliferating, undifferentiated and differentiated) keratinocytes. It is important to note that Siglec-7 may also have additional specificities i.e also recognise 2-8 and 2-3-linked moieties (59, 60) (these were not identified by our lectin array screen) as well as detecting more complex epitopes, beyond isolated glycans (61). Interestingly, recent genome-wide CRISPR-aided screen highlighted the importance of additional enzymes (62) for generaton of Siglec-7 ligands, including C1GALT1. This makes sense as C1GALT1 is a core extension enzyme acting at the beginning of the synthesis pathway which generates core of the O-glycans exposed on the sEV surface, hence required as an anchor for the exposed ST6GAL1-generated moieties added subsequently. Recent study by Büll et al (61) indicated that the core extension feature prevails over the capping glycan features and the binding is completely abolished in the C1GALT1C1 (C1GLAT1-specific chaperone; COSMC) knockout. Hence, given the role of O-glycosylation for Siglec-7 recognition, it seems that the changes in the expression of this enzyme that we noted upon analysis of data from both the epidermal samples and cytokine-stimulated keratinocytes (normal (35) and FLG insufficient) may also critically contribute to the observed effect. It is unclear, however, if the direct product(s) of the enzyme (T-antigen) may be detected by Siglec-7; further glycan modifications by sequential enzymatic action of additional capping enzymes are likely required for the recognition; this is where ST6GAL1 may execute its role in our system as moiety exposed as a part of complex Siglec-7-recognized epitopes; additional role of sulfation has to be also considered given that a group of carbohydrate sulfotransferases (CHSTs) has been implicated in the binding affinity for both Siglecs we found important for sEV-cell interactions (61, 63). Some moieties may be of course less involved, e.g. our negative blocking data for MMR receptor which has high affinity to Gal1β-3GalNAc glycans (47) suggest considerably lower importance of this linkage in our model. In our study we were not able to match our data to enzymes implicated in the binding of Siglec-9 ligands, ST3GAL4/6 (61). Overall, our results support the notion on the complexity in the Siglec system; e.g. in our experiments we only observed slight disruption of the cellular interaction with Siglec-2 blokage (not significant, despite shared receptor specificity to the 2-6 linkage). This may be dependent on the breadth of the accepted ligand pool which is constricted for this Siglec and the differences in affinities of specific glycans containing the linkage in comparison to that of Siglec-7 and -9 as shown in detail by Blixt et al. (44).

Interestingly, ST6GAL1 seems to be an important enzyme during influenza infection, since the virus uses sialic acid-containing glycans as cellular entry points (64). It has been shown that ST6GAL1 expression also correlates with poor tumour prognosis (65) and affects multiple mechanisms related to cancer (66), suggesting that the immune effect is not limited to infection. ST6GAL1 was also recently shown as enzymatically active cargo of both exosome-like sEVs and exomeres, capable of transferring sialyltransferase activity to receipient cells and inducing expression of sialylated proteins on the cell membrane (67). As for the C1GALT1 enzyme, it has also been implicated in cancer; however, the role is less clear-cut, with contradicting data on the pro-/antitumorogenic effect (68). The enzyme has also been linked to IgA nephropathy by deposition of galactose-deficient IgA1 (Gd-IgA1) circulating in the patients with *C1GALT1* mutation (69).

The exact identity of the sEV-expressed proteins which may be modified by the ST6GAL1 enzyme is not known, although we identified several proteins present within keratinocyte-derived sEVs which are likely to undergo such modification. Literature indicates some potential examples in sEVs, e.g. in a study in ovarian cancer-derived vesicles galectin-3-binding protein (LGAL3BP), was previously identified as a sialoprotein (70). LGAL3BP is a known sialic acid-dependent ligand for CD33-related Siglec family (71) (including Siglec-7 and Siglec-9), so could be potentially important in the sEV-mediated communication in the skin. Similarly, β1 integrins (72), also present in our samples, could be similarly modified; lipid molecules which may also be sialylated, as shown for ganglioside GD3 delivering a direct T cell inhibitory signal *via* sialic acid (57). With respect to successful adhesion and delivery of the inhibitory signal, also the spatial distribution of sialylated proteins on the surface of keratinocyte-derived sEVs could potentially affect the outcome, especially if the proteins segregate into rafts or microdomains on the surface of sEVs to mediate specific interaction by formation of so called “glycosynapses” (73); however, we did not determine this in this study.

In summary, our study showed that in the context of AD *C. albicans* promotes sialic acid-enriched glycosylation pattern on the host sEVs to increase their interaction with inhibitory Siglec receptors. We may predict potential future applicability of targeting this glycosylation-sEV-Siglec-dependent pathway as a novel adjuvant therapy in skin candidiasis in AD patients; however, we cannot exclude potential applicability also beyond the skin.

## Materials and Methods

### Samples

Ethical approvals for the study were obtained from the Independent Bioethics Committee for Scientific Research at Medical University of Gdansk, ethical approval numbers: NKBBN/559/2017-2018 and NKBBN/621-574/2020. Perioperative skin samples (2-3 cm^2^) were obtained from the individuals undergoing surgery at the Department of Surgical Oncology, Medical University of Gdansk, Poland. Until isolation, the material was stored in PBS (Sigma-Aldrich, St. Louis, MO, USA), with 1% penicillin and streptomycin (Sigma-Aldrich, St. Louis, MO, USA), in 4°C. Buffy coats were obtained as a byproduct from blood donations coming from healthy donors at the Regional Blood Centre in Gdansk.

### Keratinocyte Isolation and Culture

Skin samples were washed in PBS with 100 U/ml penicillin + 100 µg/ml streptomycin, subcutaneous adipose tissue was removed and the samples were cut into small pieces. Epidermis was removed from the dermis after 2-3 hour incubation in dispase (12U/mL, Corning, NY, USA) at 37°C and digested in a 0.25% trypsin-EDTA solution (Sigma-Aldrich, St. Louis, MO, USA) with trypsin inhibition with EpiLife Medium supplemented with EpiLife™ Defined Growth Supplement (EDGS) (Thermo Fisher Scientific, Waltham, MA, USA), antibiotics and 10% FBS (Sigma-Aldrich, St. Louis, MO, USA). Keratinocytes were seeded in a collagen IV-coated dishes (Corning, NY, USA) in EpiLife medium supplemented with EDGS, 100 U/ml penicillin + 100 µg/ml streptomycin (Sigma-Aldrich, St. Louis, MO, USA) and 10% FBS. The next day, the medium was changed to a serum-free EpiLife with EDGS and antibiotics with regular changes every 2 days of culture at 37°C, 5% CO_2_. For experiments, pooled NHEK cultures from n=3-4 donors were used. N/TERT-1 keratinocytes obtained as a kind gift from Prof Jim Rhinwald were cultured in Keratinocyte SFM medium (Thermo Fisher Scientific, Waltham, MA, USA) + 25 µg/mL bovine pituitary extract (Thermo Fisher Scientific, Waltham, MA, USA) + 0.2 ng/mL Epidermal Growth Factor (Thermo Fisher Scientific, Waltham, MA, USA) + 0.4 mM Ca^2+^. ShC and shFLG cells were grown in Dulbecco’s Modified Eagle’s Medium (DMEM-high glucose, Sigma-Aldrich, St. Louis MO, USA). Media used for EV isolation contained no animal products or were supplemented with EV-depleted FBS.

### Calcium Switch and AD-Relevant Treatments

Upon reaching 80% confluence in a serum-free EpiLife with EDGS supplemented with antibiotics (free from animal products), cells were washed and cultured for 3 days as undifferentiated (in 0.06 mM Ca^2+^) or differentiated (in 1.5 mM Ca^2+^) cells in the following conditions: untreated or treated with atopic dermatitis (AD) cytokine cocktail (20 ng/mL IL-4, and 10 ng/mL IL-13, IL-22 and TSLP each; Peprotech, London, UK). For pathogen treatment addition of 75 ng/mL of a selected AD-relevant inactivated pathogens; *Candida albicans* (prick test; Inmunotek, Madrid, Spain) and *Staphylococcus aureus* (heat-killed) was applied.

### Heat-Killed Bacteria

1 ml of overnight culture of *S. aureus* “Newman” (2.4 x 10^9^ CFU/mL) was centrifuged at 1700 x g. The cell pellet was washed with PBS and centrifuged 1700 x g, 5 min (2x) and resuspended in 1 ml PBS, followed by heat treatment with shaking (80°C, 30 min, 1000 rpm). The resulting suspension of heat-killed bacteria was cooled on ice and protease inhibitors (final concentration: 1 µM of E-64, 0.5 µg/mL of pepstatin A and 5 µM of leupeptin) were added after heat treatment in order to retain their stability and stored in -20°C.

### EV Isolation

The exosome-enriched fraction of sEVs (100K pellet) secreted by keratinocytes was isolated by the differential ultracentrifugation protocol as in **Figure 1A**. Briefly, conditioned medium was first centrifuged at 300 x g (Megafuge 16R TX-400 centrifuge, Thermo Scientific, Waltham, MA, USA) for 10 min to remove the cellular debris, followed by 2,000 x g (Megafuge 16R TX-400 centrifuge, Thermo Scientific, Waltham, MA, USA) for 10 min to remove soluble proteins and apoptotic bodies (AP; 2K pellet). The supernatant was ultracentrifuged at 10,000 x g (maximum rotation speed) for 30 min (OptimaTM L-90K or OptimaTM LE-80K ultracentrifuge, Beckman Coulter, Brea, CA, USA) to isolate microvesicles (MVs; 10K pellet). The supernatant was then ultracentrifuged at 100,000 x g (maximum rotation speed) for 16 h to pellet exosome-enriched fraction (exosome-enriched sEVs; 100K pellet). The 100K pellet was washed in PBS by additional spin and stored at -20°C for further use.

sEVs were labelled using the PKH67 Green Fluorescent Cell Linker Midi Kit for General Cell Membrane Labeling (Sigma-Aldrich, St. Louis, MO, USA) according to the manufacturer’s instructions. In brief, sEVs corresponding to 6 x 10^6^ keratinocytes for a given culture condition were resuspended in 100 μl of Diluent C and incubated with 5 μM PKH67 for 5 minutes. The labelling reaction was quenched by the addition of 2x volume of EV-depleted complete RPMI medium (Sigma-aldrich, St. Louis, MO, USA) (supplemented with 10% EV-depleted FBS and 100 U/ml penicillin + 100 µg/ml streptomycin), and samples were then washed in PBS (100,000 x g (maximum rotation speed), 16 h, 4°C). Labelled sEVs were resuspended in EV-depleted complete RPMI medium and used directly for MDDC interaction assessment.

### Western Blot

Cell lysates were prepared in RIPA buffer (Cell Signalling Technology, Danvers, MA, USA) supplemented with the cOmplete™, Mini, EDTA-free Protease Inhibitor Cocktail (Sigma-Aldrich, St. Louis, MO, USA), vortexed well and centrifuged for 15 min at 4°C in 13,000 rpm. The supernatant was collected, and 4X Bolt™ LDS Sample Buffer (Thermo Fisher Scientific, Waltham, MA, USA) (10x diluted) was added. The same amount of the loading buffer was added to the EV samples in PBS (10x diluted). The samples were heated for 10 min at 80°C. EV samples (an equivalent of EVs isolated from 1.71 mln cells per well) were then loaded onto the Bolt™ 4 to 12% Bis-Tris precast gel (Thermo Fisher Scientific, Waltham, MA, USA) and ran for 30-60 min at 150V and then transferred onto nitrocellulose membranes (iBlot™ 2 Transfer Stacks, Thermo Fisher Scientific, Waltham, MA, USA) in the iBlot transfer system (iBlot™ 2 Gel Transfer Device, Thermo Fisher Scientific, Waltham, MA, USA). Next, the membranes were blocked in 5% fat-removed powdered milk (Carl Roth, Karlsruhe, Germany) in PBS for an hour on the shaker, and next incubated with primary antibodies overnight at 4°C on the shaker (all primary Abs were diluted 1:250, only CD63 was 1:500 diluted). The next day membranes were washed 3x for 5 min in PBS-T (PBS + 0.5ml/l Tween 20) and secondary antibodies (LI-COR Biosciences, Lincoln, NE, USA) in PBS-T (1:25,000) were added. After 30 min of incubation with the secondary antibodies the membranes were washed 3x for 5 min in PBS-T and once in PBS, paper-dried and scanned using the Odyssey CLx Near Infrared Imaging System (LI-COR Biosciences, Lincoln, NE, USA).

### EV Characterisation

Electron microscopy imaging was carried out as a paid service by the University of Gdansk Electron Microscopy Facility. Briefly, samples were adsorbed onto formvar/carbon-coated copper grids size 300 mesh (EM Resolutions, Sheffield, UK), stained with 1.5% uranyl acetate (BD Chemicals Ltd.), and imaged by Tecnai electron microscope (Tecnai Spirit BioTWIN, FEI, Hillsboro, OR, USA). Nanoparticle Tracking Analysis was carried out using NS300 NanoSight NTA (Malvern Panalytical, Malvern, UK), the EV samples were diluted 1000x in PBS.

#### Dendritic Cell Models

PBMCs were separated from buffy coat samples with Lymphoprep (STEMCELL Technologies, Vancouver, BC, Canada). CD14^+^ cells were isolated by the MojoSort™ Human CD14 Selection Kit (BioLegend, San Diego, CA, USA) according to the manufacturer’s protocol. Cells were seeded in 24-well plates in complete RPMI medium (supplemented with 10% heat-inactivated FBS and 100 U/ml penicillin + 100 µg/ml streptomycin) at a density of 1 x 10^6^ cells in 1 ml of medium per well. Cells were cultured for 7 days at 37°C and 5% CO_2_ in the presence of 50 ng/mL (500 U/mL) GM-CSF and 200 ng/mL (1000 U/mL) IL-4 for the generation of immature monocyte-derived dendritic cells (iMDDCs). Cytokine-supplemented medium was replaced on day 2 and 4 of the culture. To generate mature monocyte-derived dendritic cells (mMDDCs) 1 μg/ml LPS (Sigma-Aldrich, St. Louis, MO, USA) was added on day 6 of the culture.

#### EV Cell Interaction Assessment and Blocking

On day 7, iMDDCs and mMDDCs were washed, resuspended in EV-depleted complete RPMI medium and seeded on 96-well round-bottom plates at a density of 0.066 x 10^6^ cells/well. Cells were then incubated for 4 h in a total volume of 100 μl/well with PKH67-labelled sEVs obtained from 1 x 10^6^ of keratinocytes cultured as previously described. Cells were then washed with PBS (10 min, 300 x g), fixed in 4% formaldehyde (Sigma-Aldrich, St. Louis, MO, USA) and the cell interaction of sEVs by MDDCs was analyzed by flow cytometry using the Millipore Guava EasyCyte Flow Cytometer (Merck Millipore, Burlington, MA, USA).

For the blocking experiment with a THP-1-based model, N/TERT-1-derived sEVs were used. sEVs were isolated by differential centrifugation as described before and quantified using NanoSight NS300 NTA (Malvern Panalytical, Malvern, UK). Before use sEVs were resuspended in Diluent C and labelled with PKH67 Green Fluorescent Cell Linker Kit (Sigma-Aldrich, St. Louis, MO, USA), for a mock control Diluent C alone was used for labelling. THP-1 cells were differentiated by culturing them in the presence of 1000 U/mL IL-4 (PeproTech, London, UK) and 50 ng/mL GM-CSF (PeproTech, London, UK) for 7 days. On days 2 and 4 of the culture the whole medium was replaced; fresh medium was supplemented with cytokines as before. On day 7 cells were collected, washed, and treated with CD206, CD207, CD209, Siglec-2, Siglec-7 or Siglec-9 antibodies (Biolegend, San Diego, CA, USA) at 10 µg/mL for 1 hour at 37°C. Next, cells were washed twice with PBS and exposed to either PKH67-labelled N/TERT-1-derived sEVs or mock control, and incubated for 4 hours at 37°C. Cells were then washed, fixed Bioligand in 4% Formaldehyde (Sigma-Aldrich, St. Louis, MO, USA) and analyzed using the Millipore Guava EasyCyte Flow Cytometer (Merck Millipore, Burlington, MA, USA).

#### Lectin Array

Lectin array 70 product (GA-Lectin-70-14) was purchased from RayBiotech (Peachtree Corners, GA, USA) and the assay was run according to the manufacturer’s protocol, with an anjustment of using PHK67-labelled sEVs directly as the sample source with the omission of flouorophore-conjugated detection antibody. The array slide was imaged with the Amersham Typhoon RGB scanner (Cy2 525BP20 filter) (Marlborough, MA, USA) at adjusted PMT voltages (intensities).

#### Mass Spectrometry

After lysis of sEVs with 1% SDS and cysteine residues’ reduction with dithiothreitol, samples were processed in a standard Multi-Enzyme Digestion Filter Aided Sample Preparation (MED-FASP) procedure with cysteine alkylation by iodoacetamide and consecutive proteolytic digestion by LysC, trypsin, and chymotrypsin. Peptides obtained after each digestion were separately desalted on a C18 resin in a STAGE Tips procedure, and subsequently measured in the data-dependent acquisition mode on a Triple TOF 5600+ mass spectrometer (SCIEX, Farmingham, MA, USA) coupled with an Ekspert MicroLC 200 Plus System (Eksigent Technologies, Redwood City, CA, USA). All measurement files were subjected to joint database search in the MaxQuant 1.6.2.6a against the Homo sapiens SwissProt database (version from 09.11.2020). Resulting intensities were normalized using Total Protein Approach and protein concentrations in pmol/mg were calculated. Concentrations were imported into Perseus software and log2-transformed, data was restricted to 50% valid values, missing values were imputed from normal distribution and all values were normalized by z-score. T-tests between the test groups were conducted, and the results with p-value lower than 0.05 were considered to be statistically significant. The mass spectrometry proteomics data have been deposited to the ProteomeXchange Consortium *via* the PRIDE partner repository with the dataset identifier: PXD031729.

#### Microarray

For the microarray study, shC and shFLG cells were grown to 80% confluence and then exposed to IL-4 and IL-13 (Peprotech, London, UK) at 50 ng/mL for the incubation time of 24 h. RNA was extracted with RNeasy kit (Qiagen, Hilden, Germany) according to the manufacturer’s instructions. The microarray was performed by ServiceXS (Leiden, Netherlands) on an Illummina HT12v4 BeadArray platform (Illumina, San Diego, CA, USA) and the data were normalized using lumi (74) and analysed with LIMMA (75). The microarray dataset has been deposited to the Gene Expression Omnibus (GEO) repository and assigned the accession number: GSE203409.

#### Analysis

Data was analysed by Graph Pad Prism version 7 with one-way ANOVA (Holm-Sidak correction); *p<0.05; **p<0.01; ***p<0.001; ****p<0.0001. Cell adhesion-related proteins in sEVs were identified using the Gene Ontology tool, available at http://geneontology.org/ (76, 77). Interactions between the proteins of interest were identified using the STRING (78) database available in the Cytoscape 3.8.2 software (https://cytoscape.org/) (79) *via* the stringApp. Glycoproteins and glycosylation-relevant enzymes within NHEKs sEVs MS dataset were identified by literature search. Glycosylation-related pathways were identified using the Reactome Pathway Database (https://reactome.org/). For the STRING analysis protein lists were subjected to STRING database analysis (78). Generated networks were obtained with confidence mode of display of network edges. As a source of interactions between proteins we used “textmining”, “experiments” and “databases” only with medium confidence interaction score (0.4) applied. Networks were not further expanded. Graphical adjustment was done using Cytoscape software platform. Single cell data on protein expression in skin population was obtained from the Human Developmental Cell Atlas available at https://developmentcellatlas.ncl.ac.uk/.

Transcriptomic data from Esaki et al. (GSE120721) was analyzed using the GEO2R tool available through the Gene Expression Omnibus (GEO) database (80, 81).

In datasets analyzed for the expression of glycosylation enzymes, i.e. Esaki et al., Leung et al. and He et al. p-values were adjusted using the Benjamini & Hochberg method.

## Data Availability Statement

The datasets presented in this study can be found in online repositories. The names of the repository/repositories and accession number(s) can be found below:

https://www.ebi.ac.uk/pride/archive/, PXD031729.

https://www.ncbi.nlm.nih.gov/geo/, GSE203409.

## Ethics Statement

The studies involving human participants were reviewed and approved by Independent Bioethics Committee for Scientific Research at Medical University of Gdansk. The patients/participants provided their written informed consent to participate in this study.

## Author Contributions

AKo, JF, ABi, LH, ABog performed experiments, analysed data and contributed to the writing and figure preparation. AKr and MD performed experiments. JL performed data analysis. JZ provided surgical samples. SG, GSO, MP interpreted the data and participated in manuscript writing. DG-O provided funding, planned experiments and analysed the data, wrote the first and subsequent paper drafts. All authors contributed to the article and approved the submitted version.

## Funding

This project has received funding from the European Union’s Horizon 2020 research and innovation programme under the Marie Skłodowska-Curie grant agreement No. 665778, as a part of POLONEZ Fellowship from the National Science Centre, Poland, UMO-2016/23/P/NZ6/04056 and from the POIR.04.04.00-00-21FA/16-00 project, carried out within the First TEAM programme of the Foundation for Polish Science co-financed by the European Union under the European Regional Development Fund and from Medical Research Council UK.

**Figure:**
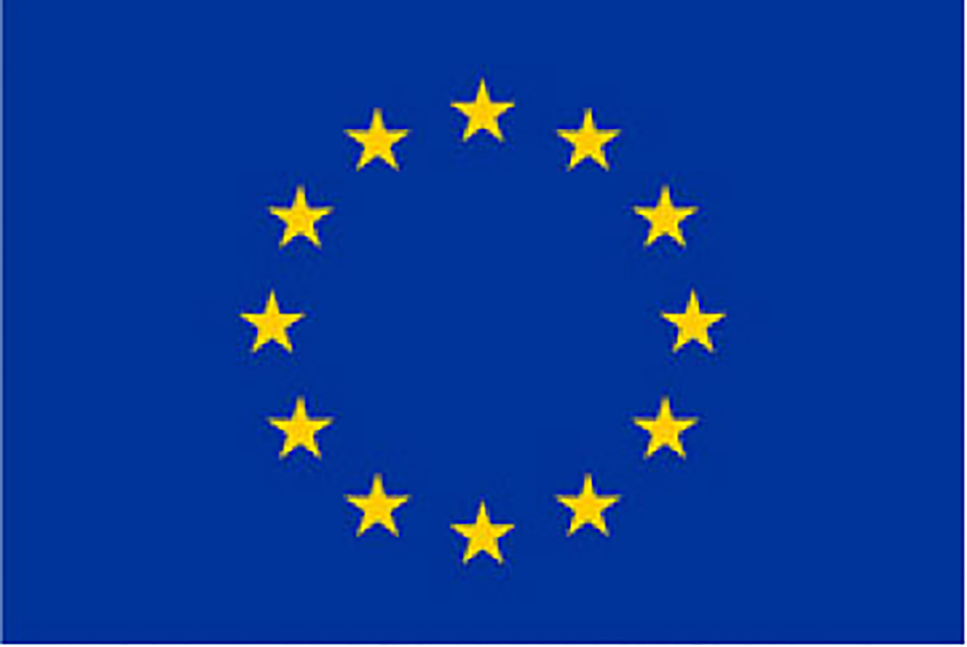


## Conflict of Interest

The authors declare that the research was conducted in the absence of any commercial or financial relationships that could be construed as a potential conflict of interest.

## Publisher’s Note

All claims expressed in this article are solely those of the authors and do not necessarily represent those of their affiliated organizations, or those of the publisher, the editors and the reviewers. Any product that may be evaluated in this article, or claim that may be made by its manufacturer, is not guaranteed or endorsed by the publisher.

## References

[1] Morita E Hide M Yoneya Y Kannbe M Tanaka A Yamamoto S. An Assessment of the Role of Candida Albicans Antigen in Atopic Dermatitis. J Dermatol (1999) 26:282–7. doi: 10.1111/j.1346-8138.1999.tb03473.x 10380428

[2] Sonesson A Bartosik J Christiansen J Roscher I Nilsson F Schmidtchen A. Sensitization to Skin-Associated Microorganisms in Adult Patients With Atopic Dermatitis Is of Importance for Disease Severity. Acta Dermato-Venereologica (2013) 93(3):340–5. doi: 10.2340/00015555-1465 23073977

[3] Hernández-Santos N Gaffen SL. Th17 Cells in Immunity to Candida Albicans. Cell Host Microbe (2012) 11(5):425–35. doi: 10.1016/j.chom.2012.04.008 PMC3358697 22607796

[4] Lin L Ibrahim AS Xu X Farber JM Avanesian V Baquir B. Th1-Th17 Cells Mediate Protective Adaptive Immunity Against Staphylococcus Aureus and Candida Albicans Infection in Mice. PloS Pathog (2009) 5(12):e1000703. doi: 10.1371/journal.ppat.1000703 20041174 PMC2792038

[5] Savolainen J Kosonen J Lintu P Viander M Pène J Kalimo K. Candida Albicans Mannan-and Protein-Induced Humoral, Cellular and Cytokine Responses in Atopic Dermatitis Patients. Clin Exp Allergy (1999) 29(6):824–31. doi: 10.1046/j.1365-2222.1999.00555.x 10336600

[6] Davidson L Van denReek JMPA Bruno M van Hunsel F Herings RMC Matzaraki V. Risk of Candidiasis Associated With Interleukin-17 Inhibitors: A Real-World Observational Study of Multiple Independent Sources. Lancet Regional Health - Europe (2022) 13:100266. doi: 10.1016/j.lanepe.2021.100266 34950923 PMC8671639

[7] Milovanovic M Drozdenko G Weise C Babina M Worm M. Interleukin-17A Promotes IgE Production in Human B Cells. J Invest Dermatol (2010) 130:2621–8. doi: 10.1038/jid.2010.175 20596087

[8] Khosravi AR Naseri Bandghorai A Moazzeni M Shokri H Mansouri P Mahmoudi M. Evaluation of Candida Albicans Allergens Reactive With Specific IgE in Asthma and Atopic Eczema Patients. Mycoses (2009) 52:326–33. doi: 10.1111/j.1439-0507.2008.01599.x 18705661

[9] Tanaka M Aiba S Takahashi K Tagami H. Reduced Proliferative Responses of Peripheral Blood Mononuclear Cells Specifically to Candida Albicans Antigen in Patients With Atopic Dermatitis–Comparison With Their Normal Reactivity to Bacterial Superantigens. Arch Dermatol Res (1996) 288:495–9. doi: 10.1007/BF02505243 8874741

[10] Gauglitz GG Callenberg H Weindl G Korting HC. Host Defence Against Candida Albicans and the Role of Pattern-Recognition Receptors. Acta Dermato-Venereologica (2012) 92:291–8. doi: 10.2340/00015555-1250 22170181

[11] Gutowska-Owsiak D Ogg GS. The Epidermis as an Adjuvant. J Invest Dermatol (2012) 132:940–8. doi: 10.1038/jid.2011.398 22217742

[12] Lyrio ECD Campos-Souza IC Corrêa LCD Lechuga GC Verícimo M Castro HC. Interaction of Mycobacterium Leprae With the HaCaT Human Keratinocyte Cell Line: New Frontiers in the Cellular Immunology of Leprosy. Exp Dermatol (2015) 24(7):536–42. doi: 10.1111/exd.12714 25828729

[13] Liu W Hsu DK Chen HY Yang RY Carraway KL 3rd Isseroff RR. Galectin-3 Regulates Intracellular Trafficking of EGFR Through Alix and Promotes Keratinocyte Migration. J Invest Dermatol (2012) 132(12):2828–37. doi: 10.1038/jid.2012.211 PMC3496033 22785133

[14] Li S Kang P Zhang W Jian Z Zhang Q Yi X. Activated NLR Family Pyrin Domain Containing 3 (NLRP3) Inflammasome in Keratinocytes Promotes Cutaneous T-Cell Response in Patients With Vitiligo. J Allergy Clin Immunol (2020) 145(2):632–45. doi: 10.1016/j.jaci.2019.10.036 31756352

[15] Bryniarski K Ptak W Jayakumar A Püllmann K Caplan MJ Chairoungdua A. Antigen-Specific, Antibody-Coated, Exosome-Like Nanovesicles Deliver Suppressor T-Cell microRNA-150 to Effector T Cells to Inhibit Contact Sensitivity. J Allergy Clin Immunol (2013) 132(1):170–81. doi: 10.1016/j.jaci.2013.04.048 PMC4176620 23727037

[16] Yoon JH Ashktorab H Smoot DT Nam SW Hur H Park WS. Uptake and Tumor-Suppressive Pathways of Exosome-Associated GKN1 Protein in Gastric Epithelial Cells. Gastric Cancer (2020) 23(5):848–62. doi: 10.1007/s10120-020-01068-2 32291710

[17] Sobo-Vujanovic A Munich S Vujanovic NL. Dendritic-Cell Exosomes Cross-Present Toll-Like Receptor-Ligands and Activate Bystander Dendritic Cells. Cell Immunol (2014) 289(1-2):119–27. doi: 10.1016/j.cellimm.2014.03.016 PMC4045011 24759079

[18] Tang MKS Yue PYK Ip PP Huang RL Lai HC Cheung ANY. Soluble E-Cadherin Promotes Tumor Angiogenesis and Localizes to Exosome Surface. Nat Commun (2018) 9(1):2270. doi: 10.1038/s41467-018-04695-7 29891938 PMC5995921

[19] Kotzerke K Mempel M Aung T Wulf GG Urlaub H Wenzel D. Immunostimulatory Activity of Murine Keratinocyte-Derived Exosomes. Exp Dermatol (2013) 22:650–5. doi: 10.1111/exd.12230 24079734

[20] Than UTT Guanzon D Broadbent JA Leavesley DI Salomon C Parker TJ. Differential Expression of Keratinocyte-Derived Extracellular Vesicle Mirnas Discriminate Exosomes From Apoptotic Bodies and Microvesicles. Front Endocrinol (2018) 9:535. doi: 10.3389/fendo.2018.00535 PMC6143807 30258405

[21] Lo Cicero A Delevoye C Gilles-Marsens F Loew D Dingli F Guéré C. Exosomes Released by Keratinocytes Modulate Melanocyte Pigmentation. Nat Commun (2015) 6(1):7506. doi: 10.1038/ncomms8506 26103923 PMC4491833

[22] Cai XW Zhu R Ran L Li YQ Huang K Peng J. A Novel Non-Contact Communication Between Human Keratinocytes and T Cells: Exosomes Derived From Keratinocytes Support Superantigen-Induced Proliferation of Resting T Cells. Mol Med Rep (2017) 16(5):7032–8. doi: 10.3892/mmr.2017.7492 28901485

[23] Smirnov A Lena AM Cappello A Panatta E Anemona L Bischetti . ZNF185 Is a P63 Target Gene Critical for Epidermal Differentiation and Squamous Cell Carcinoma Development. Oncogene (2019) 38:1625–38. doi: 10.1038/s41388-018-0509-4 PMC6755960 30337687

[24] Hu G Gong A-Y Roth AL Huang BQ Ward HD Zhu G. Release of Luminal Exosomes Contributes to TLR4-Mediated Epithelial Antimicrobial Defense. PloS Pathog (2013) 9(4):e1003261. doi: 10.1371/journal.ppat.1003261 23592986 PMC3617097

[25] Nocera AL Mueller SK Stephan JR Hing L Seifert P Han X. Exosome Swarms Eliminate Airway Pathogens and Provide Passive Epithelial Immunoprotection Through Nitric Oxide. J Allergy Clin Immunol (2019) 143(4):1525–1535.e1. doi: 10.1016/j.jaci.2018.08.046 30442371

[26] Lässer C O'Neil SE Shelke GV Sihlbom C Hansson SF Gho YS. Exosomes in the Nose Induce Immune Cell Trafficking and Harbour an Altered Protein Cargo in Chronic Airway Inflammation. J Trans Med (2016) 14(1):181. doi: 10.1186/s12967-016-0927-4 PMC4913423 27320496

[27] Chavez-Munoz C Morse J Kilani R Ghahary A. Primary Human Keratinocytes Externalize Stratifin Protein *via* Exosomes. J Cell Biochem (2008) 104(6):2165–73. doi: 10.1002/jcb.21774 18452139

[28] Chavez-Muñoz C Kilani RT Ghahary A. Profile of Exosomes Related Proteins Released by Differentiated and Undifferentiated Human Keratinocytes. J Cell Physiol (2009) 221(1):221–31. doi: 10.1002/jcp.21847 19530224

[29] Théry C Witwer KW Aikawa E Alcaraz MJ Anderson JD Andriantsitohaina R. Minimal Information for Studies of Extracellular Vesicles 2018 (MISEV2018): A Position Statement of the International Society for Extracellular Vesicles and Update of the MISEV2014 Guidelines. J Extracellular Vesicles (2018) 7(1):1535750. doi: 10.1080/20013078.2018.1535750 30637094 PMC6322352

[30] Schjoldager KT Narimatsu Y Joshi HJ Clausen H. Global View of Human Protein Glycosylation Pathways and Functions. Nat Rev Mol Cell Biol (2020) 21:729–49. doi: 10.1038/s41580-020-00294-x 33087899

[31] Esaki H Ewald DA Ungar B Rozenblit M Zheng X Xu H. Identification of Novel Immune and Barrier Genes in Atopic Dermatitis by Means of Laser Capture Microdissection. J Allergy Clin Immunol (2015) 135(1):153–63. doi: 10.1016/j.jaci.2014.10.037 PMC4452382 25567045

[32] Leung DYM Calatroni A Zaramela LS Lebeau PK Dyjack N Brar K. The Nonlesional Skin Surface Distinguishes Atopic Dermatitis With Food Allergy as a Unique Endotype. Sci Trans Med (2019) 11(480):eaav2685. doi: 10.1126/scitranslmed.aav2685 PMC7676854 30787169

[33] He H Bissonnette R Wu J Diaz A Saint-Cyr Proulx E Maari C. Tape Strips Detect Distinct Immune and Barrier Profiles in Atopic Dermatitis and Psoriasis. J Allergy Clin Immunol (2021) 147(1):199–212. doi: 10.1016/j.jaci.2020.05.048 32709423

[34] Reynolds G Vegh P Fletcher J Poyner EFM Stephenson E Goh I. Developmental Cell Programs are Co-Opted in Inflammatory Skin Disease. Science (2021) 371(6527):eaba6500. doi: 10.1126/science.aba6500 33479125 PMC7611557

[35] Amano W Nakajima S Kunugi H Numata Y Kitoh A Egawa G. The Janus Kinase Inhibitor JTE-052 Improves Skin Barrier Function Through Suppressing Signal Transducer and Activator of Transcription 3 Signaling. J Allergy Clin Immunol (2015) 136(3):667–77.e7. doi: 10.1016/j.jaci.2015.03.051 26115905

[36] Kezic S Jakasa I. Filaggrin and Skin Barrier Function. Curr Problems Dermatol (2016) 49:1–7. doi: 10.1159/000441539 26844893

[37] Brown SJ McLean WHI. One Remarkable Molecule: Filaggrin. J Invest Dermatol (2012) 132(3 Pt 2):751–62. doi: 10.1038/jid.2011.393 PMC3378480 22158554

[38] Wang XW Wang JJ Gutowska-Owsiak D Salimi M Selvakumar TA Gwela A. Deficiency of Filaggrin Regulates Endogenous Cysteine Protease Activity, Leading to Impaired Skin Barrier Function. Clin Exp Dermatol (2017) 42(6):622–31. doi: 10.1111/ced.13113 28556377

[39] Gutowska-Owsiak D Salimi M Selvakumar TA Wang X Taylor S Ogg GS. Histamine Exerts Multiple Effects on Expression of Genes Associated With Epidermal Barrier Function. J Investigational Allergology Clin Immunol (2014) 24(4):231–9. 25219105

[40] Marwah I Wang X Chan H Ogg GS Gutowska-Owsiak D. Filaggrin-Insufficiency in Keratinocytes Influences Responsiveness of Allergen-Specific T Cells to Cognate Antigen and Compounds Barrier Function Deficiency. Clin Immunol (2014) 153(1):153–5. doi: 10.1016/j.clim.2014.04.011 24786917

[41] Shimoda A Tahara Y Sawada SI Sasaki Y Akiyoshi K. Glycan Profiling Analysis Using Evanescent-Field Fluorescence-Assisted Lectin Array: Importance of Sugar Recognition for Cellular Uptake of Exosomes From Mesenchymal Stem Cells. Biochem Biophys Res Commun (2017) 491(3):701–7. doi: 10.1016/j.bbrc.2017.07.126 28751214

[42] Dickson MA Hahn WC Ino Y Ronfard V Wu JY Weinberg RA. Human Keratinocytes That Express hTERT and Also Bypass a P16(INK4a)-Enforced Mechanism That Limits Life Span Become Immortal Yet Retain Normal Growth and Differentiation Characteristics. Mol Cell Biol (2000) 20(4):1436–47. doi: 10.1128/MCB.20.4.1436-1447.2000 PMC85304 10648628

[43] Smits JPH Niehues H Rikken G van Vlijmen-Willems IMJJ van deZande GWHJF Zeeuwen PLJM. Immortalized N/TERT Keratinocytes as an Alternative Cell Source in 3D Human Epidermal Models. Sci Rep (2017) 7(1):11838. doi: 10.1038/s41598-017-12041-y 28928444 PMC5605545

[44] Blixt O Collins BE van denNieuwenhof IM Crocker PR Paulson JC. Sialoside Specificity of the Siglec Family Assessed Using Novel Multivalent Probes: Identification of Potent Inhibitors of Myelin-Associated Glycoprotein. J Biol Chem (2003) 278(33):31007–19. doi: 10.1074/jbc.M304331200 12773526

[45] Ibarlucea-Benitez I Weitzenfeld P Smith P Ravetch JV. Siglecs-7/9 Function as Inhibitory Immune Checkpoints *In Vivo* and Can Be Targeted to Enhance Therapeutic Antitumor Immunity. Proc Natl Acad Sci USA (2021) 118(26):e2107424118. doi: 10.1073/pnas.2107424118 34155121 PMC8256000

[46] Lübbers J Rodríguez E van Kooyk Y. Modulation of Immune Tolerance *via* Siglec-Sialic Acid Interactions. Front Immunol (2018) 9:2807. doi: 10.3389/fimmu.2018.02807 30581432 PMC6293876

[47] Functional Glycomics Gateway. Glycan-Binding Specificities of CLEC2, BDCA2, Langerin, DEC205, Endo180, MMR and DCAL-1 (2006). Available at: http://www.functionalglycomics.org/glycomics/HServlet?operation=view&sideMenu=no&psId=primscreen_PA_v2_411_0329200 (Accessed April 11, 2022).

[48] Hovhannisyan L Czechowska E Gutowska-Owsiak D. The Role of Non-Immune Cell-Derived Extracellular Vesicles in Allergy. Front Immunol (2021) 12:702381. doi: 10.3389/fimmu.2021.702381 34489951 PMC8417238

[49] Robbins PD Morelli AE. Regulation of Immune Responses by Extracellular Vesicles. Nat Rev Immunol (2014) 14(3):195–208. doi: 10.1038/nri3622 24566916 PMC4350779

[50] Williams C Pazos R Royo F González E Roura-Ferrer M Martinez A. Assessing the Role of Surface Glycans of Extracellular Vesicles on Cellular Uptake. Sci Rep (2019) 9(1):11920. doi: 10.1038/s41598-019-48499-1 31417177 PMC6695415

[51] Biedermann B Gil D Bowen DT Crocker PR. Analysis of the CD33-Related Siglec Family Reveals That Siglec-9 Is an Endocytic Receptor Expressed on Subsets of Acute Myeloid Leukemia Cells and Absent From Normal Hematopoietic Progenitors. Leukemia Res (2007) 31(2):211–20. doi: 10.1016/j.leukres.2006.05.026 16828866

[52] Walter RB Raden BW Zeng R Häusermann P Bernstein ID Cooper JA. ITIM-Dependent Endocytosis of CD33-Related Siglecs: Role of Intracellular Domain, Tyrosine Phosphorylation, and the Tyrosine Phosphatases, Shp1 and Shp2. J Leukocyte Biol (2008) 83(1):200–11. doi: 10.1189/jlb.0607388 17947393

[53] Delaveris CS Chiu SH Riley NM Bertozzi CR. Modulation of Immune Cell Reactivity With Cis-Binding Siglec Agonists. Proc Natl Acad Sci USA (2021) 118(3):e2012408118. doi: 10.1073/pnas.2012408118 33431669 PMC7826350

[54] Varchetta S Brunetta E Roberto A Mikulak J Hudspeth KL Mondelli MU. Engagement of Siglec-7 Receptor Induces a Pro-Inflammatory Response Selectively in Monocytes. PloS One (2012) 7(9):e45821. doi: 10.1371/journal.pone.0045821 23029261 PMC3461047

[55] Perdicchio M Ilarregui JM Verstege MI Unger WWJ. Sialic Acid-Modified Antigens Impose Tolerance *via* Inhibition of T-Cell Proliferation and *De Novo* Induction of Regulatory T Cells. Proc Natl Acad Sci USA (2016) 113(12):3329–34. doi: 10.1073/pnas.1507706113 PMC4812702 26941238

[56] Clayton K Vallejo AF Davies J Sirvent S Polak ME. Langerhans Cells-Programmed by the Epidermis. Front Immunol (2017) 8:1676. doi: 10.3389/fimmu.2017.01676 29238347 PMC5712534

[57] Shenoy GN Loyall J Berenson CS Kelleher RJ Jr Iyer V Balu-Iyer SV. Sialic Acid–Dependent Inhibition of T Cells by Exosomal Ganglioside GD3 in Ovarian Tumor Microenvironments. J Immunol (2018): 201(12):3750–58. doi: 10.4049/jimmunol.1801041 PMC6289713 30446565

[58] Chung LM Liang JA Lin CL Sun LM Kao CH. Cancer Risk in Patients With Candidiasis: A Nationwide Population-Based Cohort Study. Oncotarget (2017) 8(38):63562–73. doi: 10.18632/oncotarget.18855 PMC5609943 28969011

[59] Yamaji T Teranishi T Alphey MS Crocker PR Hashimoto Y. A Small Region of the Natural Killer Cell Receptor, Siglec-7, Is Responsible for Its Preferred Binding to α2,8-Disialyl and Branched α2,6-Sialyl Residues: A Comparison With Siglec-9. J Biol Chem (2002) 277(8):6324–32. doi: 10.1074/jbc.M110146200 11741958

[60] Alphey MS Attrill H Crocker PR van Aalten DMF. High Resolution Crystal Structures of Siglec-7: Insights Into Ligand Specificity in the Siglec Family. J Biol Chem (2003) 278(5):3372–7. doi: 10.1074/jbc.M210602200 12438315

[61] Büll C Nason R Sun L Narimatsu Y. Probing the Binding Specificities of Human Siglecs by Cell-Based Glycan Arrays. Proc Natl Acad Sci USA (2021) 118(17):e2026102118. doi: 10.1073/pnas.2026102118 33893239 PMC8092401

[62] Wisnovsky S Möckl L Malaker SA Bertozzi CR. Genome-Wide CRISPR Screens Reveal a Specific Ligand for the Glycan-Binding Immune Checkpoint Receptor Siglec-7. Proc Natl Acad Sci USA (2021) 118(5):e2015024118. doi: 10.1073/pnas.2015024118 33495350 PMC7865165

[63] Jung J Enterina JR Bui DT Mozaneh F Lin P-H Nitin. Carbohydrate Sulfation As a Mechanism for Fine-Tuning Siglec Ligands. ACS Chem Biol (2021) 16(11):2673–89. doi: 10.1021/acschembio.1c00501 34661385

[64] Broszeit F Tzarum N Zhu X Nemanichvili N Eggink D Leenders T. N-Glycolylneuraminic Acid as a Receptor for Influenza A Viruses. Cell Rep (2019) 27(11):3284–94. doi: 10.1016/j.celrep.2019.05.048 PMC6750725 31189111

[65] Liu N Zhu M Linhai Y Song Y Gui X Tan G. Increasing HER2 A2,6 Sialylation Facilitates Gastric Cancer Progression and Resistance *via* the Akt and ERK Pathways. Oncol Rep (2018) 40(5):2997–3005. doi: 10.3892/or.2018.6680 30226606

[66] Garnham R Scott E Livermore KE Munkley J. ST6GAL1: A Key Player in Cancer. Oncol Lett (2019) 18(2):983–9. doi: 10.3892/ol.2019.10458 PMC6607188 31423157

[67] Zhang Q Higginbotham JN Jeppesen DK Yang YP Li W McKinley ET. Transfer of Functional Cargo in Exomeres. Cell Rep (2019) 27(3):940–54. doi: 10.1016/j.celrep.2019.01.009 PMC6559347 30956133

[68] Sun X Zhan M Sun Xu Liu W Meng X. C1GALT1 in Health and Disease (Review). Oncol Lett (2021) 22(2):589. doi: 10.3892/ol.2021.12850 34149900 PMC8200938

[69] Gale DP Molyneux K Wimbury D Higgins P Levine AP Caplin B. Galactosylation of IgA1 Is Associated With Common Variation in C1GALT1. J Am Soc Nephrol (2017) 28(7):2158–66. doi: 10.1681/ASN.2016091043 PMC5491291 28209808

[70] Escrevente C Grammel N Kandzia S Zeiser J Tranfield EM Conradt HS. Sialoglycoproteins and N-Glycans From Secreted Exosomes of Ovarian Carcinoma Cells. PloS One (2013) 8(10):e78631. doi: 10.1371/journal.pone.0078631 24302979 PMC3840218

[71] Läubli H Alisson-Silva F Stanczak MA Siddiqui SS Deng L Verhagen A. Lectin Galactoside-Binding Soluble 3 Binding Protein (LGALS3BP) Is a Tumor-Associated Immunomodulatory Ligand for CD33-Related Siglecs. J Biol Chem (2014) 289(48):33481–91. doi: 10.1074/jbc.M114.593129 PMC4246102 25320078

[72] Seales EC Jurado GA Brunson BA Wakefield JK Frost AR Bellis SL. Hypersialylation of Beta1 Integrins, Observed in Colon Adenocarcinoma, may Contribute to Cancer Progression by Up-Regulating Cell Motility. Cancer Res (2005) 65(11):4645–52. doi: 10.1158/0008-5472.CAN-04-3117 15930282

[73] Cohen M Varki A. The Sialome-Far More Than the Sum of its Parts. Omics A J Integr Biol (2010) 14(4):455–64. doi: 10.1089/omi.2009.0148 20726801

[74] Du P Kibbe WA Lin SM. Lumi: A Pipeline for Processing Illumina Microarray. Bioinformatics (2008) 24(13):1547–8. doi: 10.1093/bioinformatics/btn224 18467348

[75] Smyth GK. Linear Models and Empirical Bayes Methods for Assessing Differential Expression in Microarray Experiments. Stat Appl Genet Mol Biol (2004) 3:3. doi: 10.2202/1544-6115.1027 16646809

[76] Ashburner M Ball CA Blake JA Botstein D Butler H Cherry JM. Gene Ontology: Tool for the Unification of Biology. The Gene Ontology Consortium. Nat Genet (2000) 25(1):25–9. doi: 10.1038/75556 PMC3037419 10802651

[77] Carbon S Douglass E Good DM Unni DR Harris NL Mungall CJ. The Gene Ontology Resource: Enriching a GOld Mine. Nucleic Acids Res (2021) 49(D1):D325–34. doi: 10.1093/nar/gkaa1113 PMC7779012 33290552

[78] Szklarczyk D Gable AL Lyon D Junge A Wyder S Huerta-Cepas J. STRING V11: Protein-Protein Association Networks With Increased Coverage, Supporting Functional Discovery in Genome-Wide Experimental Datasets. Nucleic Acids Res (2019) 47(D1):D607–13. doi: 10.1093/nar/gky1131 PMC6323986 30476243

[79] Shannon P Markiel A Ozier O Baliga NS Wang JT Ramage D. Cytoscape: A Software Environment for Integrated Models of Biomolecular Interaction Networks. Genome Res (2003) 13(11):2498–504. doi: 10.1101/gr.1239303 PMC403769 14597658

[80] Barrett T Wilhite SE Ledoux P Evangelista C Kim IF Tomashevsky M. NCBI GEO: Archive for Functional Genomics Data Sets–Update. Nucleic Acids Res (2013) 41:D991–5. doi: 10.1093/nar/gks1193 PMC3531084 23193258

[81] Edgar R Domrachev M Lash AE. Gene Expression Omnibus: NCBI Gene Expression and Hybridization Array Data Repository. Nucleic Acids Res (2002) 30(1):207–10. doi: 10.1093/nar/30.1.207 PMC99122 11752295

