# Supplementary figures and images for: Exposure of Keratinocytes to Candida Albicans in the Context of Atopic Milieu Induces Changes in the Surface Glycosylation Pattern of Small Extracellular Vesicles to Enhance Their Propensity to Interact With Inhibitory Siglec Receptors
Source: Front Immunol. 2022 Jun 9;13:884530. doi: 10.3389/fimmu.2022.884530 (PMC9248261; doi:10.3389/fimmu.2022.884530)

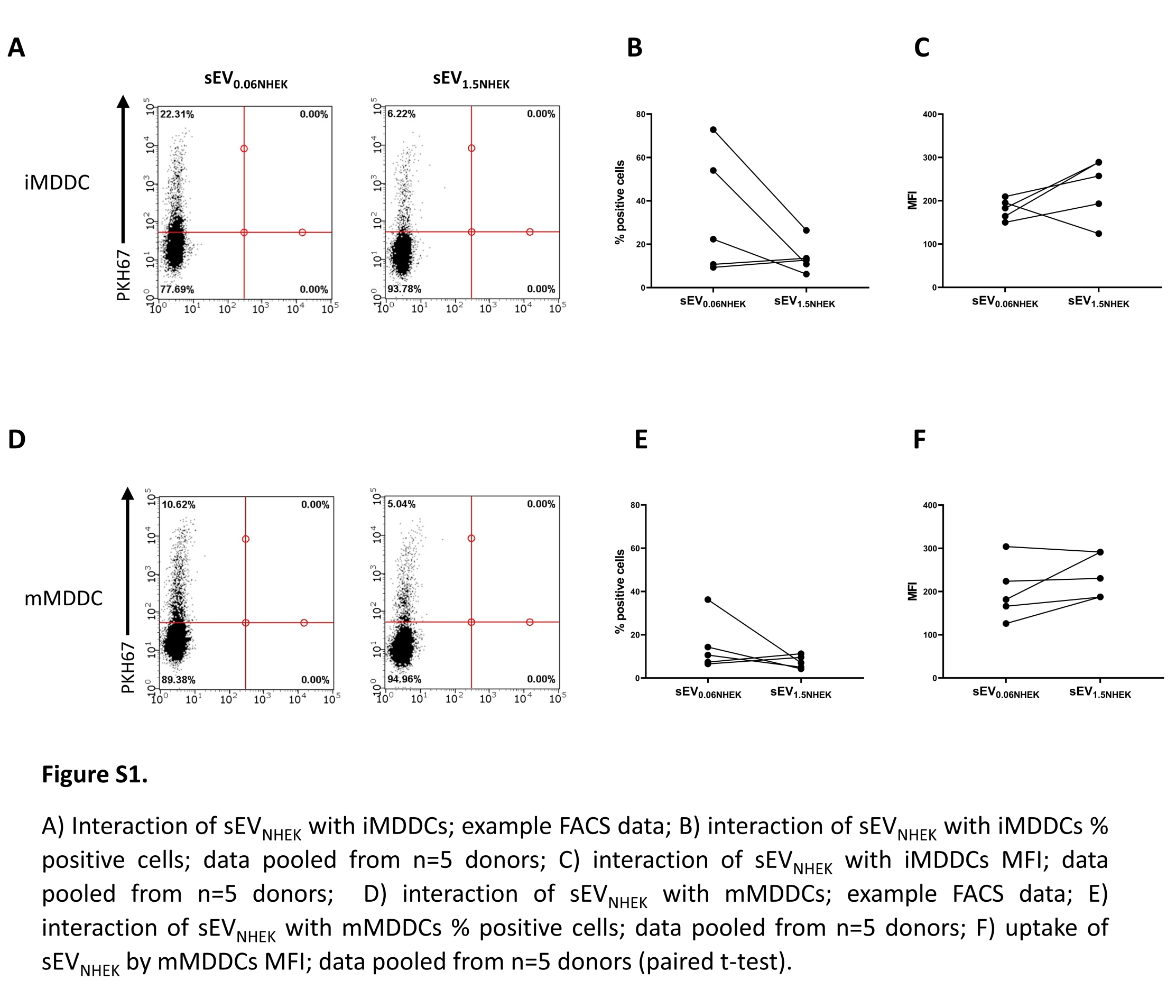

Supplement: Supplementary file 1 [file Image_1.jpeg]

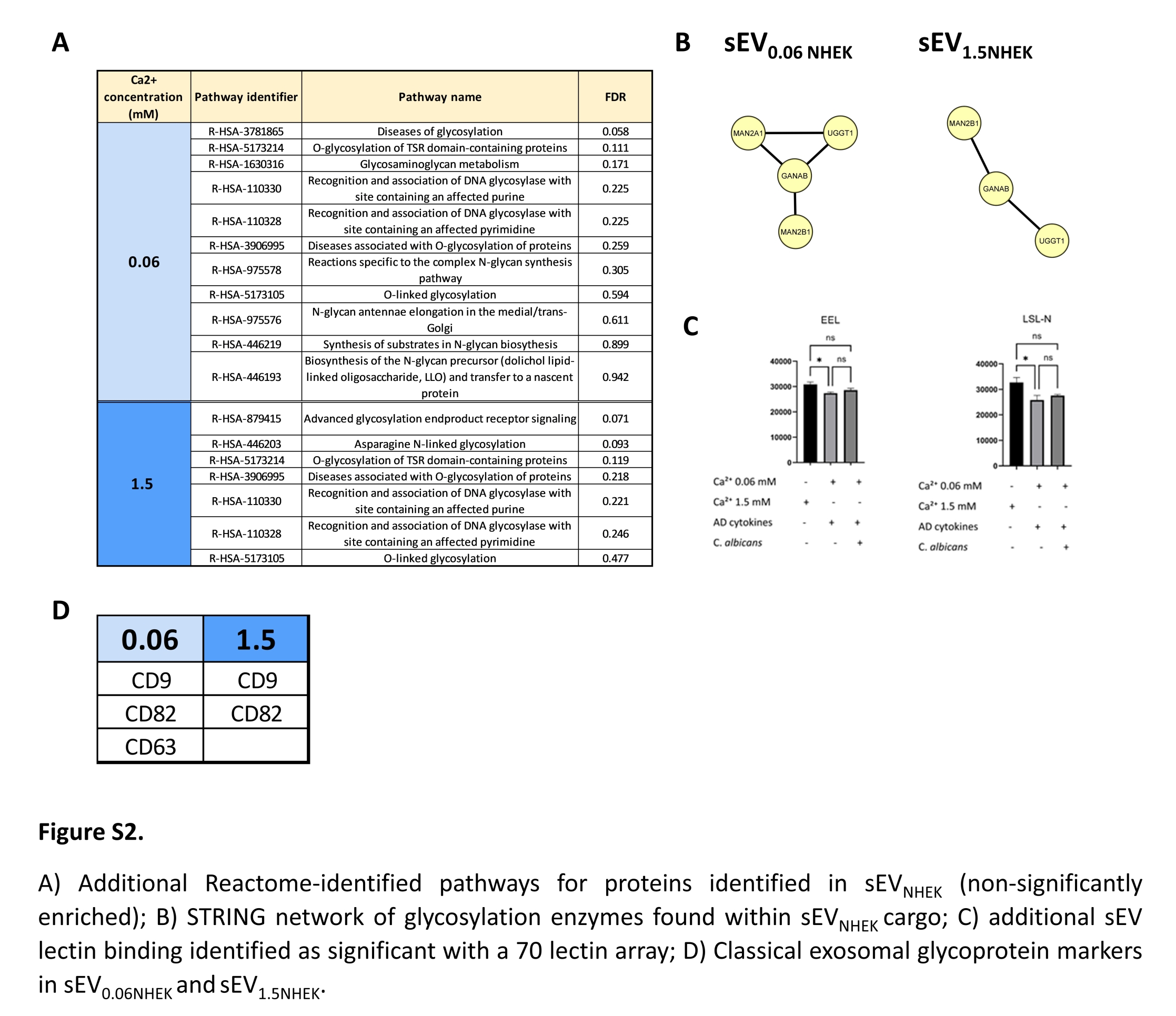

Supplement: Supplementary file 2 [file Image_2.jpeg]

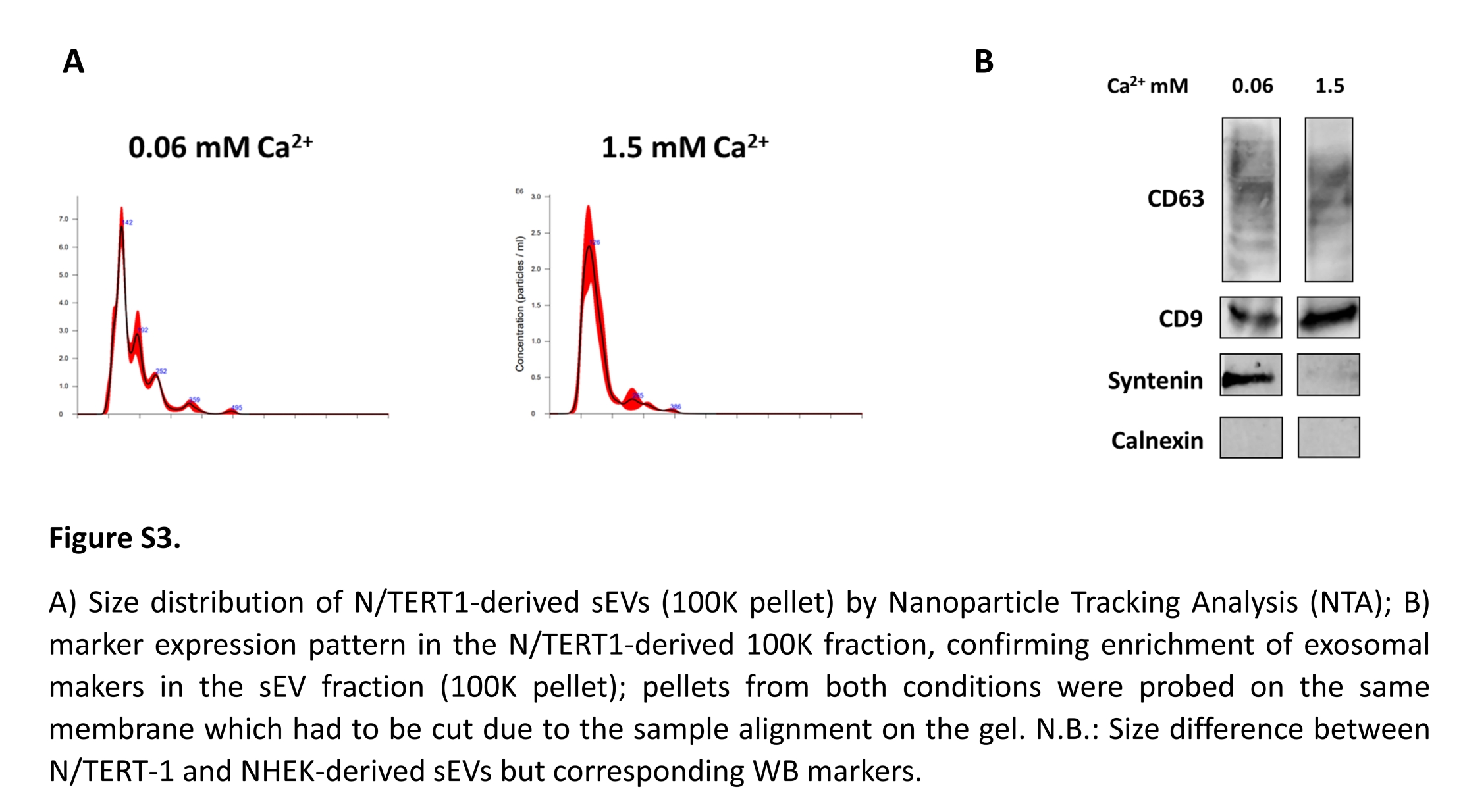

Supplement: Supplementary file 3 [file Image_3.jpeg]

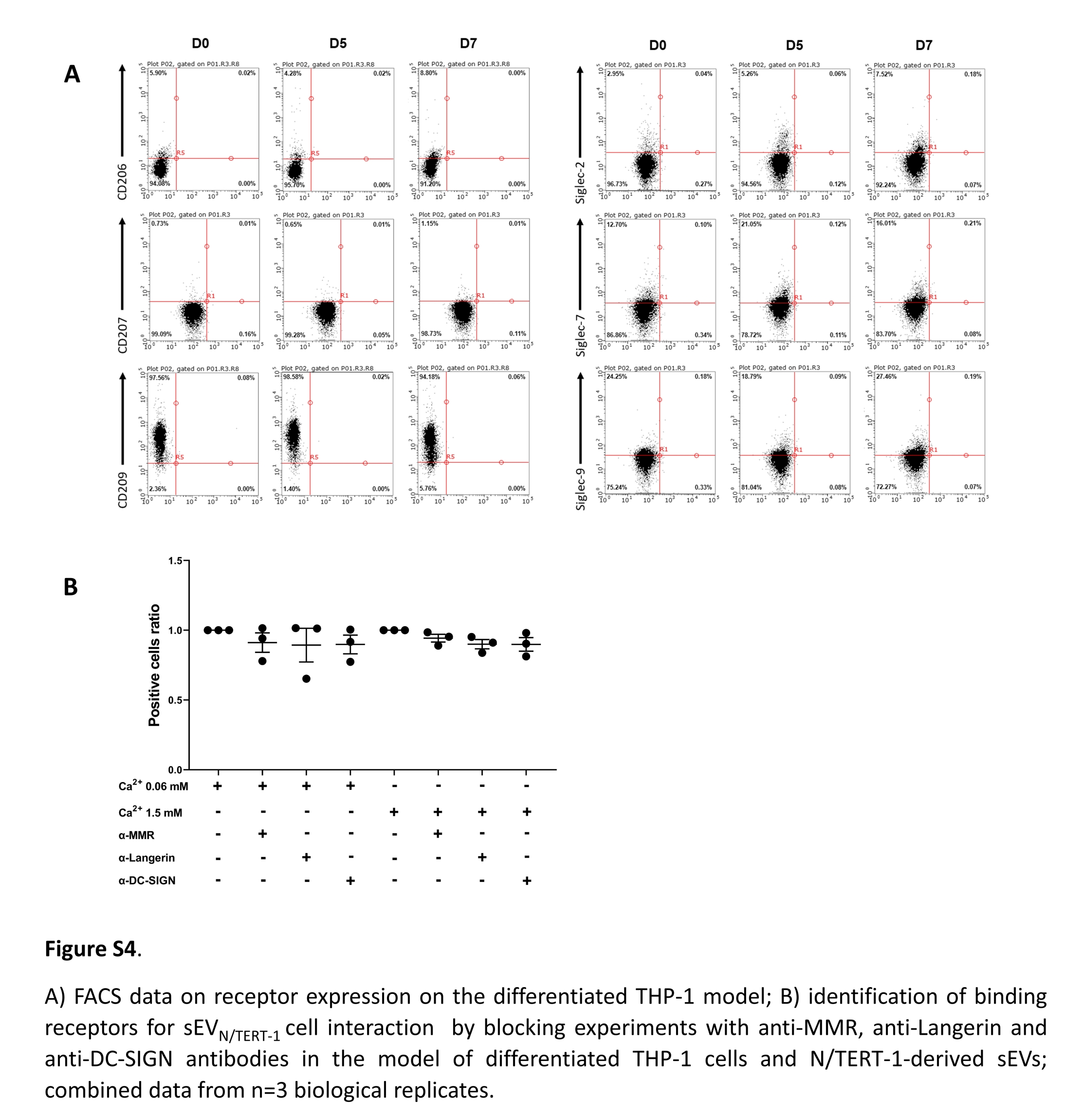

Supplement: Supplementary file 4 [file Image_4.jpeg]

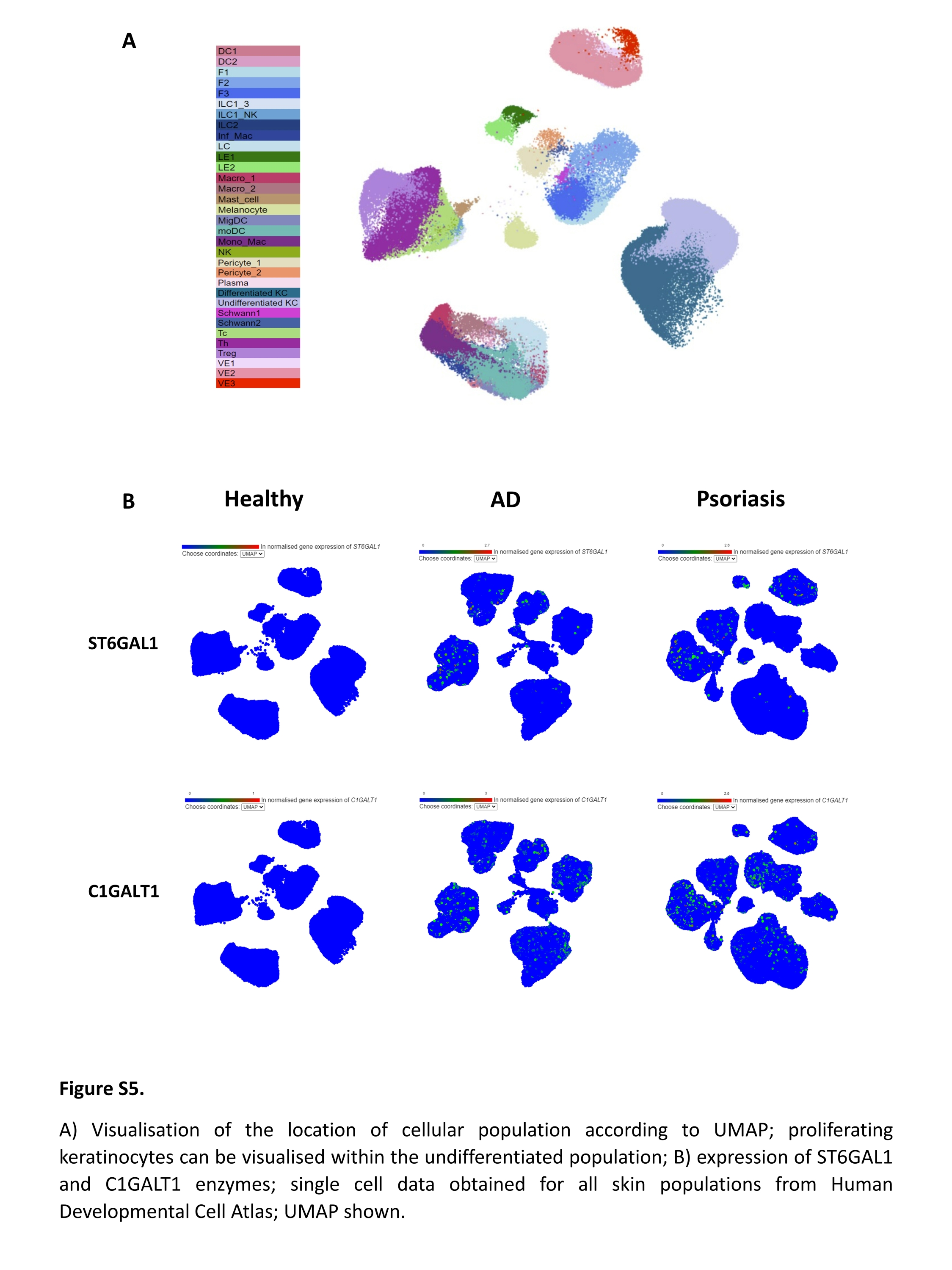

Supplement: Supplementary file 5 [file Image_5.jpeg]

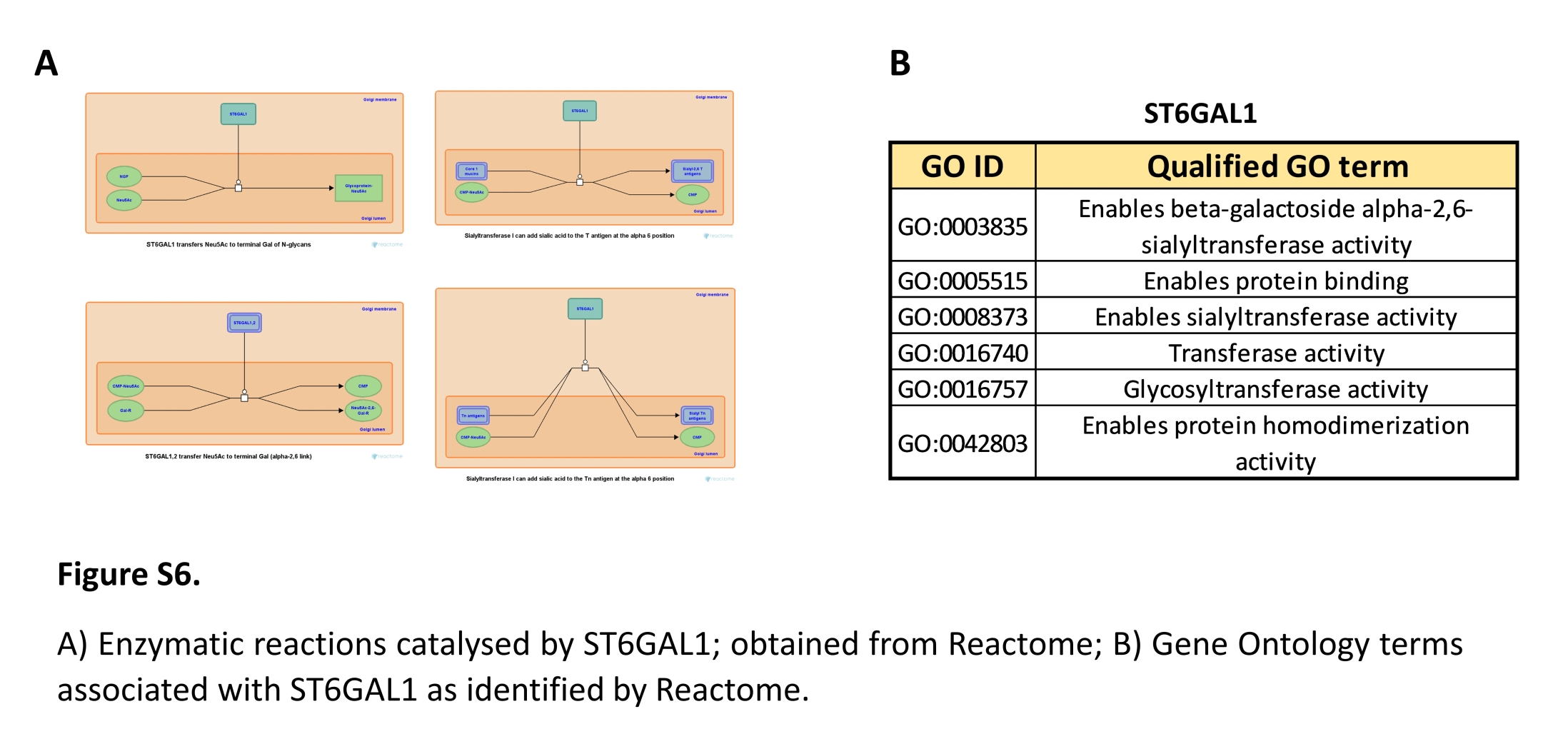

Supplement: Supplementary file 6 [file Image_6.jpeg]
