# Supplementary material for: Exposure of Keratinocytes to Candida Albicans in the Context of Atopic Milieu Induces Changes in the Surface Glycosylation Pattern of Small Extracellular Vesicles to Enhance Their Propensity to Interact With Inhibitory Siglec Receptors
Source: Front Immunol. 2022 Jun 9;13:884530. doi: 10.3389/fimmu.2022.884530 (PMC9248261; doi:10.3389/fimmu.2022.884530)
Supplement: Supplementary file 9 [file Table_3.docx]

**Table S3.** Full table of carbohydrate moiety/substrate/product-specificity of glycosylation enzymes identified in the network linked to C. albicans-specific PRRs, including references.

| **Glycosylation Enzyme** | **Carbohydrate moiety added** | **Substrate** | **Product** | **Recognizing DC PRR** |
| --- | --- | --- | --- | --- |
| LARGE xylosyl- and glucuronyltransferase (LARGE) | Xylα1-3GlcAβ1- | αDG | [Xylα1-3GlcAβ1-]αDG^1^ | ? |
| Glycoprotein-N-acetylgalactosamine 3-beta-galactosyltransferase 1 (C1GALT1) | Gal | GalNAc | Galβ1-3GalNAc^2^ | MMR ^3^ |
| Alpha-1,6-mannosylglycoprotein 6-beta-N-acetylglucosaminyltransferase A (MGAT5) | GlcNAc | αMan | GlcNAcβ1-6Manα1-6^4^ | ? |
| Beta-1,4-galactosyltransferase 1 (B4GALT1) | Gal | GlcNAc | Galβ1-4GlcNAc^5^ | ? |
| Lactosylceramide 4-alpha-galactosyltransferase (A4GALT) | Gal | Galβ1-4GlcCer | Galα1-4Galβ1-4GlcCer^6^ | ? |
| Core 1 beta3-galactosyltransferase-specific molecular chaperone (C1GALT1C1) | Gal | GalNAc | Galβ1-3GalNAc^7^ | MMR ^3^ |
| Alpha-(1,3)-fucosyltransferase 4 (FUT4) | Fuc | GlcNAc | Fucα1-3GlcNAc^8^ | ? |
| N-acetyllactosaminide beta-1,6-N-acetylglucosaminyl-transferase (GCNT2) | GlcNAc | Galβ1-4 | GlcNAcβ1-6Galβ1-4^9^ | ? |
| Galactosylgalactosylxylosylprotein 3-beta-glucuronosyltransferase 1 (B3GAT1) | GlcA | Galβ1-3 | GlcAβ1-3Galβ1-3^6^ | ? |
| Beta-galactoside alpha-2,6-sialyltransferase 1 (ST6GAL1) | Neu5Ac | Gal | Neu5Acα2-6Gal^10^ | Siglec 1 ^11,12^  SIglec 7 ^11,12^  Siglec 9 ^11,12,13^ |
| Alpha-2,8-sialyltransferase 8B (ST8SIA2) | Neu5Ac | Neu5Ac | Neu5Acα2-8Neu5Ac ^14^ | Siglec 1 ^11,12^  Siglec 5 ^11,12^  Siglec 7 ^11,12^  Siglec 9 ^11,12,13^ |

**Abbreviations**

**GlcA** = Glucuronic acid; **GlcCer** = Glucosylceramide; **Xyl** = Xylose; **αDG** = Alpha dystroglycan; **LARGE** = The like acetylglucosaminyltransferase; **DC** = Dendritic cell; **PRR** = Pattern recognition receptor; **GalNAc** = N-acetylglucosamine; **GlcNAc** = N-acetylglucosamine; **Fuc** = Fucose; **Gal** = Galactose; **Neu5Ac** = N-acetylneuraminic acid; **Man** = Mannose; **MMR** = Macrophage mannose receptor; **Siglec** = sialic acid-binding immunoglobulin-type of lectin

**References**

1. Inamori, K. I. *et al.* Dystroglycan Function Requires Xylosyl- and Glucuronyltransferase Activities of LARGE. *Science* **335**, 93 (2012).
2. Ju, T., Brewer, K., Souza, A. D., Cummings, R. D. & Canfield, W. M. Cloning and expression of human core 1 beta1,3-galactosyltransferase. *J Biol Chem* **277**, 178–186 (2002).
3. Raman, R. *et al.* Advancing glycomics: Implementation strategies at the consortium for functional glycomics. *Glycobiology* **16**, 82–90 (2006).
4. Park, C., Jin, U. H., Lee, Y. C., Cho, T. J. & Kim, C. H. Characterization of UDP-N-acetylglucosamine:alpha-6-d-mannoside beta-1,6-N-acetylglucosaminyltransferase V from a human hepatoma cell line Hep3B. *Arch Biochem Biophys* **367**, 281–288 (1999).
5. Ramasamy, V. *et al.* Oligosaccharide preferences of beta1,4-galactosyltransferase-I: crystal structures of Met340His mutant of human beta1,4-galactosyltransferase-I with a pentasaccharide and trisaccharides of the N-glycan moiety. *J Mol Biol* **353**, 53–67 (2005).
6. Kojima, Y. *et al.* Molecular cloning of globotriaosylceramide/CD77 synthase, a glycosyltransferase that initiates the synthesis of globo series glycosphingolipids. *Journal of Biological Chemistry* **275**, (2000).
7. Ju, T. & Cummings, R. D. A unique molecular chaperone Cosmc required for activity of the mammalian core 1 β3-galactosyltransferase. *Proceedings of the National Academy of Sciences* **99**, 16613–16618 (2002).
8. Weston, B. W., Nair, R. P., Larsen, R. D. & Lowe, J. B. Isolation of a novel human alpha (1,3)fucosyltransferase gene and molecular comparison to the human Lewis blood group alpha (1,3/1,4)fucosyltransferase gene. Syntenic, homologous, nonallelic genes encoding enzymes with distinct acceptor substrate specificities. *Journal of Biological Chemistry* **267**, 4152–4160 (1992).
9. Bierhuizen, M. F. A., Mattei, M. G. & Fukuda, M. Expression of the developmental I antigen by a cloned human cDNA encoding a member of a beta-1,6-N-acetylglucosaminyltransferase gene family. *Genes & Development* **7**, 468–478 (1993).
10. Kuhn, B. *et al.* The structure of human α-2,6-sialyltransferase reveals the binding mode of complex glycans. *Acta Crystallogr D Biol Crystallogr* **69**, 1826–1838 (2013).
11. Angata, T. & Brinkman-Van der Linden, E. C. M. I-type lectins. *Biochimica et Biophysica Acta (BBA) - General Subjects* **1572**, 294–316 (2002).
12. Monaco, G. *et al.* RNA-Seq Signatures Normalized by mRNA Abundance Allow Absolute Deconvolution of Human Immune Cell Types. *Cell Rep* **26**, 1627-1640.e7 (2019).
13. Crocker, P. R., Paulson, J. C. & Varki, A. Siglecs and their roles in the immune system. *Nature Reviews Immunology 2007 7:4* **7**, 255–266 (2007).
14. Sato, C., Hane, M. & Kitajima, K. Relationship between ST8SIA2, polysialic acid and its binding molecules, and psychiatric disorders. *Biochimica et Biophysica Acta (BBA) - General Subjects* **1860**, 1739–1752 (2016).
