# Supplementary material for: Exposure of Keratinocytes to Candida Albicans in the Context of Atopic Milieu Induces Changes in the Surface Glycosylation Pattern of Small Extracellular Vesicles to Enhance Their Propensity to Interact With Inhibitory Siglec Receptors
Source: Front Immunol. 2022 Jun 9;13:884530. doi: 10.3389/fimmu.2022.884530 (PMC9248261; doi:10.3389/fimmu.2022.884530)
Supplement: Supplementary file 10 [file Table_4.docx]

**Table S4.** Full table of carbohydrate moieties enriched in the “more adhesive” sEVs as identified by lectin array, together with matched potential binding receptors on antigen presenting cells as identified by the literature search, including references.

| **Lectin** | **Carbohydrate Specificity (PAMP)** | **DC PRR** |
| --- | --- | --- |
| BC2L A | High mannose | MMR ^1–3^  Dectin 2 ^3^  DC-SIGN ^2–4^  Langerin ^2,3,5^  CLEC2 ^2^  Mincle ^6^  DCIR ^2^  BDCA2 ^7,8^ |
| Discoidin 1 | GalNAc^9^ | Langerin ^5^  MGL ^6^ |
|  | Galβ1-3GalNAc^10^ | MMR ^6^ |
|  | GalNAcβ1-3Gal^10^ | MGL ^6^ |
|  | αGalNAc (Tn antigen) | MGL ^3^ |
|  | Gal^10^ | MGL ^11^  MMR ^6^ |
|  | GlcNAcβ1-3Gal ^10^ | Langerin ^2^ |
|  | LacNAc | BDCA ^2^  DCIR ^2^  Galectin 1 ^12^ |
| F17AG | GlcNAc | Langerin ^3,13^  BDCA2 ^7,8^ |
|  | pNP-GlcNAc^14^ | ? |
|  | GlcNAcβ1-3Gal^14^ | Langerin ^6^ |
| GAL1S | β-galactoside ^12,15^ | Galectin ^12,15^ |
|  | Gal ^16^ | MGL ^11^  MMR ^6^ |
|  | LacNAc ^12,15^ | Galectin 1 ^12,15^ |
|  | branched-LacNAc | Galectin ^12,15^ |
| Gal2 | GalNAcα1-3Gal (Blood group A) | MGL ^6^  DC-SIGN ^3,17,18^ |
|  | Lac^16^ | BDCA2 ^2^  CLEC2B ^2^ |
|  | branched-LacNAc | Galectin ^12,15^ |
| Gal7s | Gal ^16^ | MGL ^11^  MMR ^6^ |
|  | Galβ1-3GlcNAc | DC-SIGN ^3,18^ |
| GS1, GSL2, BSL1 | αGal | MMR ^6^  MGL ^11^ |
|  | α3GalNAc | MGL ^6^ |
| Jacalin | Galβ1-3GalNAc | MMR ^6^ |
| MOA | Galα1-3Gal | MMR ^6^ |
|  | Galα1-3Galβ1-4GlcNAc | DC-SIGN ^3,18^ |
|  | Galα1-3Fucα1-2Galβ1-4GlcNAc (Blood group B) | DC-SIGN ^3,18^  Langerin ^3,18^ |
| MPL, MPA | Gal^19^ | MMR ^6^  MGL ^11^ |
|  | GalNAc^19^ | MGL ^6^ |
|  | Galβ1-3GalNAc | MMR ^6^ |
| PHA-L | Man^20^ | MMR ^2,5^  Langerin ^5^  DC-SIGN ^2^  BDCA2 ^7,8^ |
|  | Gal ^20^ | MGL ^11^  MMR ^6^ |
|  | GlcNAc ^20^ | Langerin ^13^  DCIR2 ^21^  BDCA2 ^7,8^ |
|  | Galβ1-4GlcNAcβ2-6GlcNAcβ1-2Manα3Manα3 | ? |
| PPL | GalNAc^22^ | MGL ^23^ |
|  | Gal^22^ | MGL ^11^  MMR ^6^ |
|  | α/βGalNAc | MGL ^6^ |
| PSL1a | Neu5Acα2-6 linkages [α2-6 Sialic acid] | Siglec 1 ^24,25^  Siglec 2 ^24,26^  Siglec 3 ^24,25^  Siglec 5 ^24,25^  Siglec 7 ^24,25^  Siglec 8 ^24,25^  Siglec 9 ^24,25,27^  Siglec 10 ^24,25^ |
|  | Neu5Acα2-6Galβ1-4GlcNAc^28^ | Siglec 1 ^25,29^  Siglec 9 ^24,25,27^ |
| Sna1 | Neu5Acα2-6Gal ^30^ | SIglec 1 ^25,27^  Siglec 7 ^25,27^  Siglec 9 ^24,25,27^ |
|  | NeuAcα2-6Galβ1-4Glc^30^ | Siglec 9 ^24,25,27^ |
|  | Neu5Acα2-6GalNAc | Siglec 3 ^24,25^  Siglec 2 ^24,26^  SIglec 5 ^24,25^  Siglec 7 ^24,25^  Siglec 9 ^24,25^ |
|  | GalNAc | MGL ^6^ |
|  | Lac | BDCA2 ^2^  CLEC2B ^2^ |
|  | GalNeu5Acα2-6Gal | ? |
| Sna2 | Gal | MMR ^6^  MGL ^11^ |
|  | GalNAc | MGL ^6^ |
|  | Neu5Acα2-6Lac^31^ | Siglec 1 ^24,25^  Siglec 2 ^24,26^  Siglec 3 ^24,25^  Siglec 5 ^24,25^  Siglec 7 ^24,25^  Siglec 8 ^24,25^  Siglec 9 ^24,25^  Siglec 10 ^24,25^ |
|  | GalNAcα1-3Gal^31^ | MGL ^6^  DC-SIGN ^3,18^ |

**Abbreviations**

**DC** = Dendritic cell; **PRR** = Pattern recognition receptor; **PAMP** = Pathogen associated molecular pattern; **LacNAc** = N-acetyllactosamine; **GalNAc** = N-acetylglucosamine; **GlcNAc** = N-acetylglucosamine; **Fuc** = Fucose; **Gal** = Galactose; **Man** = Mannose **Neu5Ac** = N-acetylneuraminic acid; **MMR** = Macrophage mannose receptor; **MGL** = Macrophage galactose type lectin; **DCIR** = Dendritic cell immunoreceptor; **BDCA2** = Blood dendritic call antigen 2; **CLEC** = C-type lectin domain family; **Siglec** = sialic acid-binding immunoglobulin-type of lectin

**References**

1. Sallusto, F., Cella, M., Danieli, C. & Lanzavecchia, A. Dendritic cells use macropinocytosis and the mannose receptor to concentrate macromolecules in the major histocompatibility complex class II compartment: downregulation by cytokines and bacterial products. *The Journal of Experimental Medicine* **182**, 389 (1995).

2. Hsu, T. L. *et al.* Profiling Carbohydrate-Receptor Interaction with Recombinant Innate Immunity Receptor-Fc Fusion Proteins. *Journal of Biological Chemistry* **284**, 34479–34489 (2009).

3. Figdor, C. G., van Kooyk, Y. & Adema, G. J. C-type lectin receptors on dendritic cells and langerhans cells. *Nature Reviews Immunology* **2**, 77–84 (2002).

4. Geijtenbeek, T. B. H. *et al.* Identification of DC-SIGN, a Novel Dendritic Cell–Specific ICAM-3 Receptor that Supports Primary Immune Responses. *Cell* **100**, 575–585 (2000).

5. Valladeau, J. *et al.* Langerin, a Novel C-Type Lectin Specific to Langerhans Cells, Is an Endocytic Receptor that Induces the Formation of Birbeck Granules. *Immunity* **12**, 71–81 (2000).

6. Raman, R. *et al.* Advancing glycomics: Implementation strategies at the consortium for functional glycomics. *Glycobiology* **16**, 82–90 (2006).

7. Jégouzo, S. A. F. *et al.* A Novel Mechanism for Binding of Galactose-terminated Glycans by the C-type Carbohydrate Recognition Domain in Blood Dendritic Cell Antigen 2. *The Journal of Biological Chemistry* **290**, 16759 (2015).

8. Lee, R. T. *et al.* Survey of immune-related, mannose/fucose-binding C-type lectin receptors reveals widely divergent sugar-binding specificities. *Glycobiology* **21**, 512–520 (2011).

9. Crowley, T. E., Nellen, W., Gomer, R. H. & Firtel, R. A. Phenocopy of discoidin I-minus mutants by antisense transformation in Dictyostelium. *Cell* **43**, 633–641 (1985).

10. Mathieu, S. v, Aragão, K. s, Imberty, A. & Varrot, A. Discoidin I from Dictyostelium discoideum and interactions with oligosaccharides: specificity, affinity, crystal structures and comparison with Discoidin II. *Journal of Molecular Biology* **400**, 50–554 (2010).

11. Sakakura, M. *et al.* Carbohydrate Binding Mechanism of the Macrophage Galactose-type C-type  Lectin 1 Revealed by Saturation Transfer  Experiments. *The Journal of Biological Chemistry* **283**, 33665 (2008).

12. Vasta, G. R. *et al.* Galectins as self/non-self recognition receptors in innate and adaptive immunity: An unresolved paradox. *Frontiers in Immunology* **3**, 199 (2012).

13. Hollmig, S. T., Ariizumi, K. & Cruz, P. D. Recognition of non-self-polysaccharides by C-type lectin receptors dectin-1 and dectin-2. *Glycobiology* **19**, 568–575 (2009).

14. Lonardi, E. *et al.* Structural sampling of glycan interaction profiles reveals mucosal receptors for fimbrial adhesins of enterotoxigenic Escherichia coli. *Biology (Basel)* **2**, 894–917 (2013).

15. Vasta, G. R. Roles of galectins in infection. *Nature Reviews Microbiology 2009 7:6* **7**, 424–438 (2009).

16. Modenutti, C. P., Capurro, J. I. B., di Lella, S. & Martí, M. A. The Structural Biology of Galectin-Ligand Recognition: Current Advances in Modeling Tools, Protein Engineering, and Inhibitor Design. *Frontiers in Chemistry* **7**, (2019).

17. Valverde, P. *et al.* Molecular Insights into DC-SIGN Binding to Self-Antigens: The Interaction with the Blood Group A/B Antigens. *ACS Chemical Biology* **14**, 1660–1671 (2019).

18. Reid, M. E., Lomas-Francis, C. & Olsson, M. L. Lewis Blood Group System. *The Blood Group Antigen FactsBook* 347–359 (2012) doi:10.1016/b978-0-12-415849-8.00009-0.

19. Gómez-Santos, L., Alonso, E., Díaz-Flores, L., Madrid, J. F. & Sáez, F. J. Characterization by Lectin Histochemistry of Two Subpopulations of Parietal Cells in the Rat Gastric Glands. *Journal of Histochemistry and Cytochemistry* **65**, 261–272 (2017).

20. Lis, H., properties, N. S.-, biology, functions and applications in & 1986, undefined. Applications of lectins. *books.google.com*.

21. Nagae, M. *et al.* Recognition of Bisecting N-Acetylglucosamine: STRUCTURAL BASIS FOR ASYMMETRIC INTERACTION WITH THE MOUSE LECTIN DENDRITIC CELL INHIBITORY RECEPTOR 2*. *The Journal of Biological Chemistry* **288**, 33598 (2013).

22. T, S. *et al.* Purification, characterization, and cDNA cloning of a lectin from the mushroom Pleurocybella porrigens. *Biosci Biotechnol Biochem* **73**, 702–709 (2009).

23. Zizzari, I. G. *et al.* MGL Receptor and Immunity: When the Ligand Can Make the Difference. *Journal of Immunology Research* **2015**, (2015).

24. Angata, T. & Brinkman-Van der Linden, E. C. M. I-type lectins. *Biochimica et Biophysica Acta (BBA) - General Subjects* **1572**, 294–316 (2002).

25. Monaco, G. *et al.* RNA-Seq Signatures Normalized by mRNA Abundance Allow Absolute Deconvolution of Human Immune Cell Types. *Cell Rep* **26**, 1627-1640.e7 (2019).

26. Reineks, E. Z., Osei, E. S., Rosenberg, A., Auletta, J. & Meyerson, H. J. CD22 expression on blastic plasmacytoid dendritic cell neoplasms and reactivity of anti-CD22 antibodies to peripheral blood dendritic cells. *Cytometry Part B: Clinical Cytometry* **76B**, 237–248 (2009).

27. Crocker, P. R., Paulson, J. C. & Varki, A. Siglecs and their roles in the immune system. *Nature Reviews Immunology 2007 7:4* **7**, 255–266 (2007).

28. Tateno, H., Winter, H. & Goldstein, I. Escherichia coli and characterization of the recombinant Neu5Acalpha2, 6Galbeta1, 4GlcNAc-specific high-affinity lectin and its mutants from the mushroom Polyporus. *Biochem. J* **382**, 667–675 (2004).

29. Crocker, P. R. *et al.* Purification and properties of sialoadhesin, a sialic acid-binding receptor of murine tissue macrophages. *EMBO Journal* **10**, 1661–1669 (1991).

30. Fischer, E. & Brossmer, R. Sialic acid-binding lectins: submolecular specificity and interaction with sialoglycoproteins and tumour cells. *Glycoconjugate Journal* **12**, 707–713 (1995).

31. Kaku, H., Peumans, W. J. & Goldstein, I. J. Isolation and characterization of a second lectin (SNA-II) present in elderberry (Sambucus nigra L.) bark. *Archives of Biochemistry and Biophysics* **277**, 255–262 (1990).
